# An epigenetic gene silencing pathway selectively acting on transgenic DNA in the green alga *Chlamydomonas*

**DOI:** 10.1038/s41467-020-19983-4

**Published:** 2020-12-08

**Authors:** Juliane Neupert, Sean D. Gallaher, Yinghong Lu, Daniela Strenkert, Na’ama Segal, Rouhollah Barahimipour, Sorel T. Fitz-Gibbon, Michael Schroda, Sabeeha S. Merchant, Ralph Bock

**Affiliations:** 1 Max-Planck-Institut für Molekulare Pflanzenphysiologie, Am Mühlenberg 1, D-14476 Potsdam-Golm, Germany; 2 University of California Los Angeles, Department of Chemistry and Biochemistry, and Institute for Genomics and Proteomics, 607 Charles E. Young Dr. East, Los Angeles, CA 90095 USA; 3 University of California Los Angeles, Department of Molecular, Cell and Developmental Biology, 610 Charles E. Young Dr. South, Los Angeles, CA 90095 USA; 4 Present Address: Nanjing University of Science and Technology, Institute of Chemical Biology and Advanced Materials, Xiaolingwei Street 200, 210094 Nanjing, China; 5 Present Address: University of California Berkeley, Departments of Molecular and Cell Biology and Plant and Microbial Biology, Berkeley, CA 94720 USA; 6 Present Address: Technical University of Kaiserslautern, Molecular Biotechnology & Systems Biology, Paul-Ehrlich-Straße 23, 67663 Kaiserslautern, Germany

**Keywords:** Histone analysis, Gene expression analysis, Plant biotechnology, Epigenetics

## Abstract

Silencing of exogenous DNA can make transgene expression very inefficient. Genetic screens in the model alga *Chlamydomonas* have demonstrated that transgene silencing can be overcome by mutations in unknown gene(s), thus producing algal strains that stably express foreign genes to high levels. Here, we show that the silencing mechanism specifically acts on transgenic DNA. Once a permissive chromatin structure has assembled, transgene expression can persist even in the absence of mutations disrupting the silencing pathway. We have identified the gene conferring the silencing and show it to encode a sirtuin-type histone deacetylase. Loss of gene function does not appreciably affect endogenous gene expression. Our data suggest that transgenic DNA is recognized and then quickly inactivated by the assembly of a repressive chromatin structure composed of deacetylated histones. We propose that this mechanism may have evolved to provide protection from potentially harmful types of environmental DNA.

## Abstract (web summary)

Strong transgene suppression has been observed in *Chlamydomonas reinhardtii*, but the underlying mechanism is unknown. Here, the authors identify a sirtuin-type histone deacetylase that selectively acts on transgenic DNA to repress gene expression by assembling a repressive chromatin structure composed of deacetylated histones.

## Introduction

The unicellular ciliated green alga *Chlamydomonas reinhardtii* belongs to the plant lineage Viridiplantae. It has become an invaluable reference system for nearly all areas of plant biology and, in addition, for medical research on ciliary diseases in humans^1^. The alga also provides an attractive production host for recombinant proteins, biofuels and green chemicals^2–5^. Although *Chlamydomonas* is readily transformable, transgene expression from the nuclear genome is notoriously inefficient^6,7^ and, moreover, often unstable in that loss of transgene expression occurs with time^8,9^. A number of strategies have been pursued to overcome this serious limitation^10^, including the construction of hybrid promoters^11^, the inclusion of endogenous introns in the expression cassette^12^ and codon optimization of the foreign gene^6,13,14^. Although these strategies alleviated the problem to some extent in some cases, the expression of even standard transgenes (like the fluorescent reporters GFP and YFP) has remained very challenging. A more general solution has emerged from mutant screens for strains that show improved transgene expression properties. Two such mutant strains, UVM4 and UVM11, were isolated from a UV mutagenesis screen^15^ and have become widely used in cell biology studies and as tools for high-level transgene expression^16–19^. Enhanced transgene expression correlates with greatly increased transcript levels, suggesting that the strains harbor mutations in gene(s) that cause strong epigenetic transgene silencing in the wild type^15^.

Here we report the identification of the gene underlying the transgene expression phenotype in the *Chlamydomonas* expression strains UVM4 and UVM11. We show that the transgene-silencing pathway specifically affects exogenously introduced DNA. Identification of a histone-modifying enzyme as the key factor for silencing to occur suggests that lack of coverage with histones is the distinguishing feature that allows cells to recognize transgenic DNA and inactivate it. The silencing mechanism identified in this work also highlights strategies how transgene expression can be improved in recalcitrant species.

## Results

### Transgenic DNA is associated with transcriptionally active chromatin in UVM4 and UVM11

**Fig. 1:**
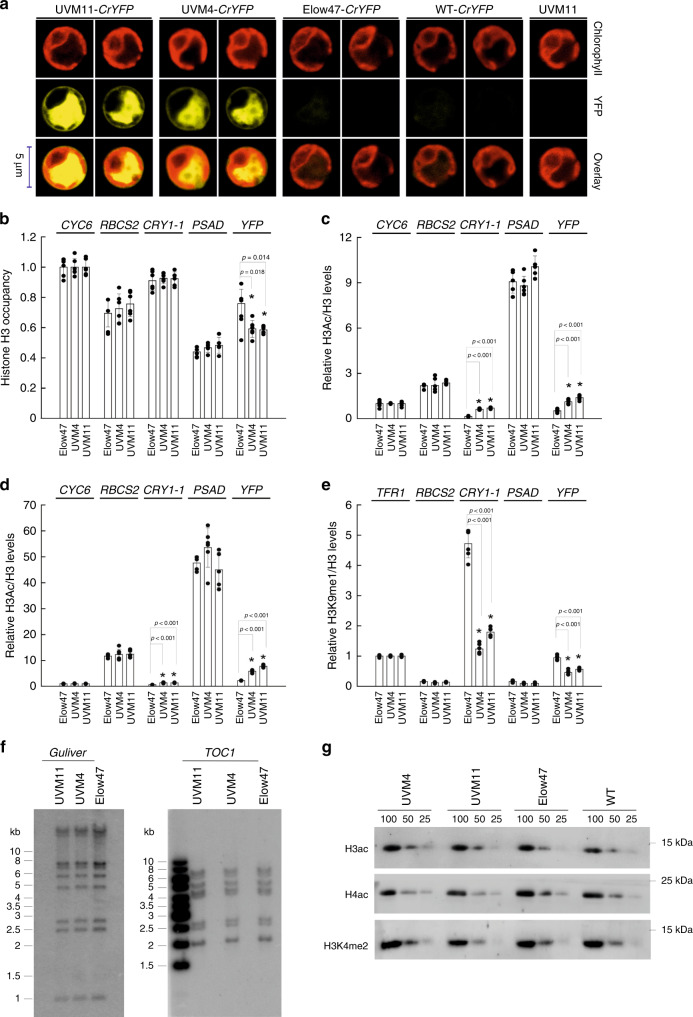
Properties of *Chlamydomonas* expression strains. **a** YFP expression capacity in the expression strains UVM4 and UVM11 and the control strains Elow47 and CC-4350 (WT). All strains were transformed with the *CrYFP* construct^14^ and analyzed by confocal laser-scanning microscopy. For each strain, two independent *CrYFP* transformants were chosen that are representative of the strength of transgene expression. Chlorophyll fluorescence, YFP fluorescence and the overlay of the two fluorescences are shown. The non-transformed strain UVM11 served as negative control. Cells from at least 20 independently transformed colonies were individually analyzed by fluorescence microscopy (at least 200 cells per colony). The images show representative cells of each strain. **b**–**e** Comparative analysis of (**b**) histone H3 occupancy, (**c**) histone H3 acetylation, (**d**) histone H4 acetylation, and (**e**) histone H3 monomethylation at lysine 9 (H3K9me1) at the transcriptional start site (TSS) region of the endogenous genes *CYC6*, *RBCS2* and *PSAD* and the transgenes *CRY1_1* and *YFP* in the expression strains (UVM4 and UVM11) and their progenitor strain Elow47. Error bars indicate the standard error of six replicates. *p < 0.05 (one-way ANOVA, Holm-Sidak post hoc test). Shown are averages and standard deviations of six ChIP experiments (individual data points shown as black dots; three experiments each were done on two independent pools of at least 1200 transformants). Values for each TSS region investigated were normalized to those obtained for the native *CYC6* promoter (**b**–**d**) or a telomere-flanking region (*TFR1*; **e**). **f** Southern blot analysis to determine the activities of the DNA transposon *Gulliver* and the retrotransposon *TOC1*, ~2 years after the isolation of the expression strains UVM4 and UVM11 from mutagenesis of Elow47. Total DNA was digested with HindIII and probed for *Gulliver* (left panel) or digested with HincII and probed for *TOC1* (right panel). Fragment sizes of the molecular weight marker are given at the left in kb. Results from this experiment (performed once) were confirmed by transposon analysis on whole-genome sequencing data (Table 2; Supplementary Data 8) **g** Immunoblot analyses to determine the levels of acetylated histone 3 (H3ac), acetylated histone 4 (H4ac), and dimethylated lysine 4 of histone 3 (H3K4me2) in the expression strains (UVM4 and UVM11) and the control strains (Elow47, and CC-4350 as WT). Total soluble protein was separated by denaturing SDS-PAGE, blotted and probed with specific antibodies as indicated. For each strain, a dilution series (100%, 50%, and 25% corresponding to 10, 5, and 2.5 µg total soluble protein, respectively) of the protein sample was loaded. Molecular weights were deduced from co-migrating protein markers and are given at the right in kDa. Each immunoblot experiment was performed at least twice with similar results obtained. Source data underlying Fig. 1b–g are provided as a Source Data file.

When transformed with the *YFP* reporter gene, strong protein accumulation is readily detectable as a very bright yellow fluorescence signal in the cytoplasm of the UVM4 and UVM11 strains (Fig. 1a). By contrast, the transformed wild-type strain (CC-4350 alias cw15-302) and the wild-type-like control strain that was used for the UV mutagenesis screen (Elow47) do not show detectable YFP fluorescence. To confirm the assumed involvement of chromatin structure in mediating the high transgene expression capacity in strains UVM4 and UVM11 (ref. ^14^), chromatin immunoprecipitation (ChIP) experiments were conducted. Using antibodies against different histone modifications with known associations to chromatin states in *Chlamydomonas*, the chromatin state at the transcriptional start site (TSS) of the transgene was assessed. Nucleosome occupancy was determined with antibodies against unmodified histone H3, active chromatin was probed with antibodies against acetylated histones H3 (K9/K14) and H4 (K5/K8/K12/K16), and repressive chromatin formation was assessed with antibodies against monomethylated lysine at position 9 of histone 3 (H3K9me1; refs. ^20,21^).

For the ChIP analysis, large pools of independent *YFP* transformants of each strain were used to compensate for position effects and other types of variation in the chromatin that are related to the transgene integration sites in the genome (see “Methods”; Fig. 1b–e). It was shown previously that, when more than 240 transformants harboring the *ble* gene were pooled, variation in *ble* expression between individual transformants was averaged out^22^. We, therefore, generated two independent pools of more than 1200 transformants in each strain background and analyzed these pools in individual ChIP experiments (Fig. 1b–e). The DNA contents of ChIPed samples were determined by triplicate quantitative real-time PCR using primers spanning the TSSs. For the euchromatic histone marks (H3ac: acetylated histone 3, and H4ac: acetylated histone 4), ChIPed DNA fragments were normalized to values obtained for the endogenous *CYC6* gene encoding cytochrome *c*_6_ (as internal standard). *CYC6* is only transcribed under copper limitation^21^ and, therefore, its chromatin state is expected to be unaltered in our strains and culture conditions. A telomere-flanking region (TFR) was shown to be associated with H3K9me1 in *Chlamydomonas* and was, therefore, chosen to normalize ChIPed DNA in our samples with an antibody targeting H3K9me1 (ref. ^21^). In addition to the TSS region of the *YFP* transgene, which is driven by a copy of the endogenous *PSAD* promoter, we also determined the chromatin status of the two strong, endogenous *RBCS2* and *PSAD* promoters, and the TSS region of the emetine resistance marker *CRY1-1* (driven by a copy of the *RBCS2* promoter sequence; vector pJN4) that had been introduced into the genome of the mutagenized strain Elow47 and served as read-out of transgene expression strength in the initial genetic screen^15^.

ChIP analysis with antibodies against the unmodified C-terminus of the core histone H3 showed that nucleosome occupancy is reduced at the transgenic TSS region (*PSAD* promoter upstream of *YFP*) in strains UVM4 and UVM11 (Fig. 1b). This finding indicates a more open chromatin structure and is consistent with increased transgene expression in UVM4 and UVM11. When specific histone marks associated with either transcriptionally active (H3ac and H4ac) or repressive (H3K9me1) chromatin were investigated, strains UVM4 and UVM11 displayed much higher levels of acetylated histones 3 and 4 at the transgenic TSS regions of both *YFP* and *CRY1-1* (Fig. 1c, d), indicating the formation of transcriptionally active chromatin at these loci. At the same time, the transgenic TSS regions are associated with significantly lower levels of the repressive histone mark H3K9me1 in UVM4 and UVM11 than in strain Elow47 (Fig. 1e), suggesting that the formation of repressive chromatin at the integrated nuclear transgenes is reduced as a consequence of the mutations in UVM4 and UVM11. Notably, no differences in histone occupancy or histone modifications were detectable at the TSS region of the endogenous *RBCS2* and *PSAD* genes, demonstrating that the changes of the chromatin state at the transgenic TSS regions have no effect on the chromatin state at the TSS of the corresponding endogenous promoter copies.

We previously showed that the loss of the transgene-suppression mechanism confers no obvious selective disadvantage to the UVM strains^15^. To test whether epigenetically controlled processes are deregulated in the mutants, we investigated whether transposons are released from suppression. To analyze copy numbers and insertion sites, Southern blot analyses were performed with mutant strains that had been continuously propagated for two years after their isolation. Two elements were investigated: *TOC1* as a retrotransposon-like element (class I), and *Gulliver* as representative of a cut-and-paste DNA transposon (class II). No changes were detected in the mutant strains in comparison to Elow47, indicating that the epigenetic regulation of transposon activity is not affected by the mutation in the transgene-suppression pathway (Fig. 1f). Similarly, sensitivity to the DNA-damaging agent zeocin was unaffected by the mutations in UVM4 and UVM11 (Supplementary Fig. 1), and both global histone occupancy and specific histone modification levels were unaltered (Fig. 1g; Supplementary Fig. 2). Together, these data suggest that the mutations in UVM4 and UVM11 affect a pathway that specifically determines chromatin structure at new genomic loci that originate from the uptake and integration of exogenous DNA. We also showed that the silencing was not affected by the methylation status of the incoming DNA in that unmethylated DNA isolated from *dam-* and *dcm-* methylation-deficient bacterial strains was silenced equally efficiently. This is consistent with previous findings that also PCR-amplified DNA undergoes efficient silencing^21^.

### Identification of the UVM11 and UVM4 mutations

**Fig. 2:**
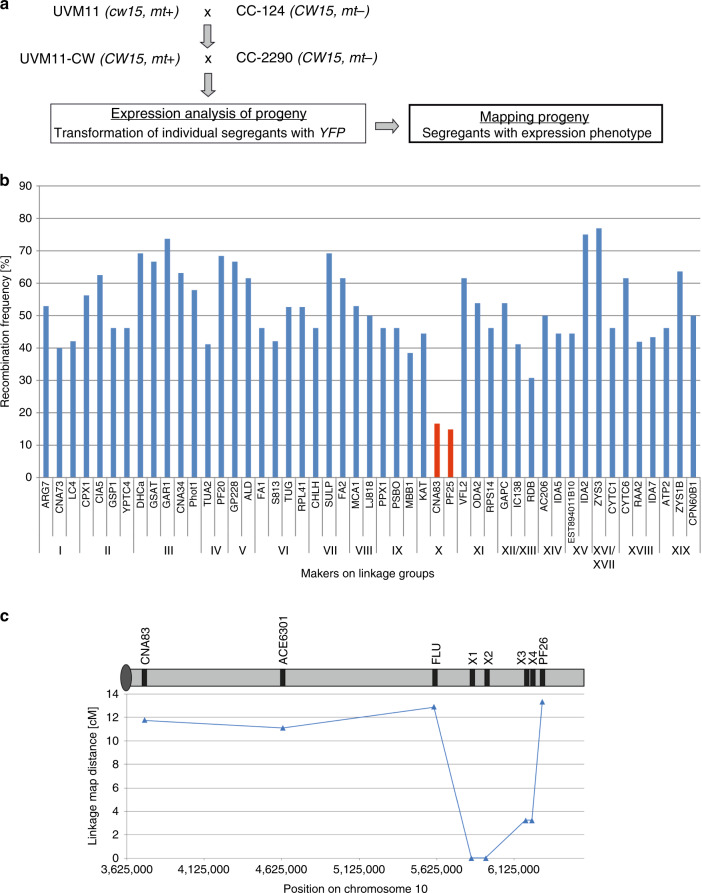
Identification of the *UVM* gene. **a** Mapping strategy. The cell wall-deficient expression strain UVM11 was crossed to the wild-type strain CC-124 in order to reintroduce cilia (required for efficient mating). Offspring (UVM11-CW) that showed similarly strong transgene expression capacity as the parental UVM11 strain was then crossed to the polymorphic strain CC-2290. The progeny of this cross was phenotyped for transgene expression efficiency by transforming every segregant with the *YFP* reporter gene and analyzing on average 20 transformants per segregant. Offspring displaying high-level YFP expression was used as mapping population. **b** Marker analysis links the transgene expression phenotype to a locus on chromosome 10. The two markers showing low recombination frequency (<20%) are indicated in red. **c** Fine mapping of *uvm* on chromosome 10. The new markers X1, X2, X3, and X4 were derived from candidate genes that reside between markers CNA83 and PF25 and potentially have chromatin-related functions^36^ (http://phytozome.jgi.doe.gov/).

To identify the mutations that cause the expression phenotype of the mutant strains, we sought to pursue a map-based cloning approach^23^. From a cross of the (cell wall-deficient, non-motile) strain UVM11 with the wild-type strain CC-124, individual segregants were screened for their capacity to express fluorescent reporter proteins to high levels. Strain UVM11-CW (*mt*+) was isolated that shows the same strong transgene expression capacity as the UVM11 parental strain, but had regained the cell wall properties and motility of the wild-type. UVM11-CW *mt*+ was crossed to the interfertile polymorphic strain CC-2290 (alias S1-D2) *mt*- (https://www.chlamycollection.org/). To be able to follow the segregation of the expression-conferring mutant alleles, a *YFP* transgene was introduced into the UVM11-CW *mt*+ strain. However, we noticed that, frequently, YFP fluorescence was retained in the entire progeny that possessed the *YFP* gene, despite the outcrossing of the expression-conferring mutation. This observation raised the possibility that, once a permissive chromatin structure has been established at the transgenic locus, the mutation in UVM11 (hereinafter referred to as *uvm* allele) is no longer needed to maintain active transgene expression. Moreover, this finding precluded the use of the YFP reporter as a phenotypic marker and, instead, necessitated phenotyping of the progeny of all crosses by transformation (i.e., transgene introduction). Thus, the individual segregants of all crosses had to be transformed with *YFP* to assess transgene expression capacity in the progeny (Fig. 2a; Supplementary Table 1; Supplementary Data 1 and 2). In total, 162 segregants were transformed at least once, and on average 20 transformed clones per segregant were screened for YFP expression, resulting in the analysis of >3240 transgenic clones. Subsequently, 48 segregants showing strong transgene expression were genotyped by PCR using marker-specific primers (http://www.chlamycollection.org/products/mapping-kits/), and recombination frequencies were calculated (Fig. 2b; Supplementary Table 2). A low recombination frequency of <20% was observed for two markers (CNA83 and PF25; Fig. 2b) on chromosome 10 (linkage group 10, LGX), indicating that the *uvm* gene resides in this region (Supplementary Data 3). The inclusion of additional markers positioned between CNA83 and PF25 (ACE6301 and FLU; Fig. 2c) confirmed this conclusion.

Next, we investigated all genes localized between the FLU and PF25 markers and identified those that are predicted to be involved in chromatin-related functions (Supplementary Data 4). We then created four new markers (X1, X2, X3, and X4) by designing primers derived from the 3′ ends of four of these genes (Cre10.g461750, Cre10.g462200, Cre10.g464264, and Cre10.g464750; Supplementary Data 4) and determined their linkage to *uvm* (Fig. 2c). Absolute linkage (in that the entire progeny displayed the marker allele associated with the UVM11-CW parent) was observed for markers X1 and X2 (Fig. 2c; Supplementary Table 2).

**Fig. 3:**
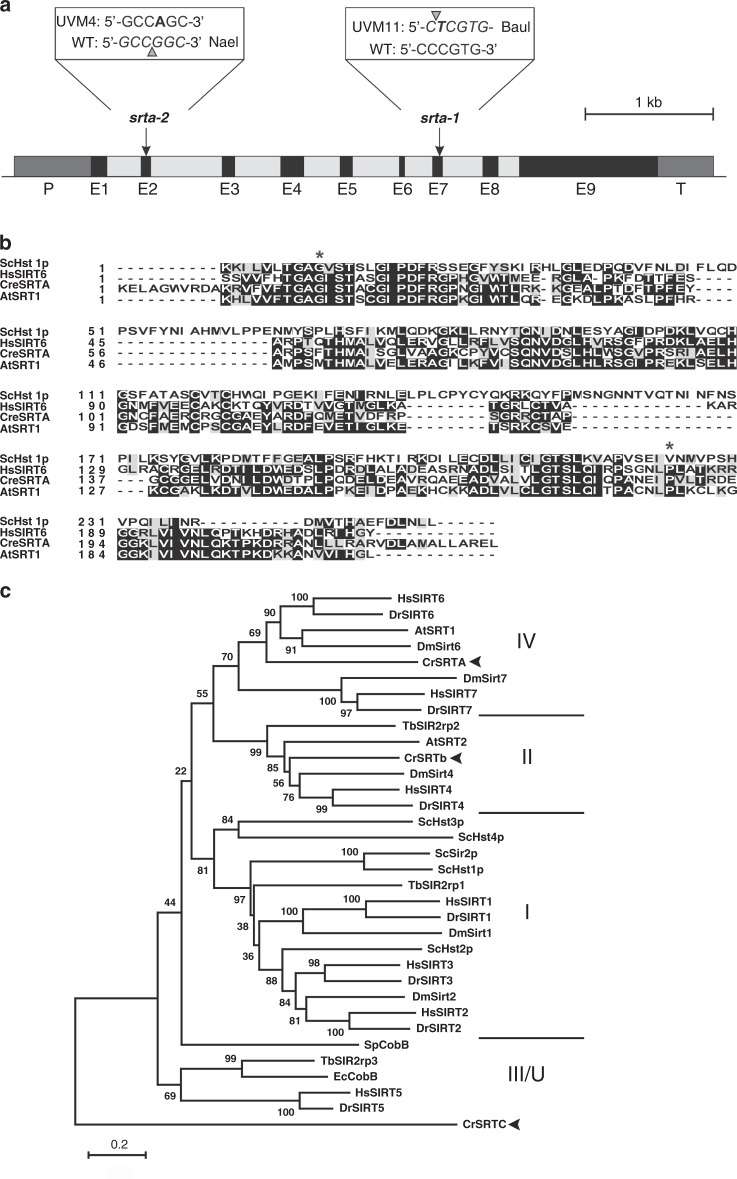
Structure of the Cre10.g462200/*UVM* gene and properties of the encoded Sir2-like NAD(+) dependent protein deacetylase. **a** Expression strains UVM4 and UVM11 each carry a non-synonymous point mutation in the coding region of the Cre10.g462200 gene. Exons (E1-E9) are shown as black, introns as light gray boxes. Promoter (P) and terminator (T) sequences are represented as dark gray boxes. Arrows indicate the positions of the point mutations in strain UVM4 (mutant allele *srta-2*) and strain UVM11 (mutant allele *srta-1*). The G-to-A transition in *srta-2* leads to the loss of a NaeI restriction site, the C-to-T transition in *srta-1* leads to gain of a BauI restriction site. **b** Alignment of the Sir2 domain of *Chlamydomonas* SRTA with the Sir2 domains of its closest homologs from *Arabidopsis* (AtSRT1), humans (HsSIRT6) and yeast (ScHst1p). Residues conserved in more than 50% of the sequences are shown in black boxes. Similar amino acids are shaded in gray. The residues affected by the two UVM mutations are marked by asterisks. **c** Phylogenetic tree of Sir2-type histone deacetylases from *Chlamydomonas reinhardtii* (CrSRTA-C) and sirtuins from other model organisms, including *Saccharomyces cerevisiae* (ScSir2p, ScHst1-4), *Drosophila melanogaster* (DmSirt1, 2, 4, 6, and 7), *Danio rerio* (DrSIRT1-7), *Homo sapiens* (HsSIRT1-7), *Arabidopsis thaliana* (AtSRT1 and AtSRT2), the protozoan *Trypanosoma brucei* (TbSIR2rp1-3), and the bacteria *Escherichia coli* (EcCobB) and *Streptococcus pneumoniae* (SpCobB). The tree is based on a ClustalW multiple sequence alignment and was conducted using the neighbor-joining method with 500 bootstrap replicates. The optimal tree with the sum of branch length = 16.60516448 is shown. The percentages of replicate trees in which the associated taxa clustered together in the bootstrap test (500 replicates) are shown next to the branches. The tree is drawn to scale, with branch lengths in the same units as those of the evolutionary distances used to infer the phylogenetic tree. The evolutionary distances were computed using the Poisson correction method, and both the ClustalW MSA and the Neighbor-Joining phylogenetic analysis were conducted with the MEGAX software (Molecular Evolutionary Genetics Analysis, https://www.megasoftware.net/mega4/). The Sir2 homologs found in *Chlamydomonas* are marked with black arrows. The sequence-based phylogenetic classes of sirtuins^26^ are given at the right (I, II, III, IV, and U).

Sequencing of the two genes (including their promoters and terminators) in strain UVM11 revealed a C-to-T transition at position +2728 of gene Cre10.g462200. Sequencing of the loci in UVM4 detected a G-to-A transition at position +431 in the same gene (Fig. 3a), strongly suggesting that Cre10.g462200 represents the *UVM* gene. To further confirm this conclusion, the genomes of both UVM strains were sequenced. Since the strains originated from UV mutagenesis, the mutation load in their genomes was high (with over 200 novel mutations per strain), as expected (Supplementary Tables 3 and 4; Supplementary Data 5). However, the number of genes containing distinct non-synonymous mutations in both strains was low and locus Cre10.g462200 was identified as one of only two such loci (and the only one on chromosome 10; Supplementary Table 5).

Cre10.g462200/*UVM* encodes a 65 kDa putative Sir2-type histone deacetylase (SRTA). The Sir2 domain of *Chlamydomonas* SRTA is highly similar to the Sir2 domains of histone deacetylases in other eukaryotes, including *Arabidopsis* (AtSRT1), humans (HsSIRT6) and yeast (ScHst1p; Fig. 3b). The Gly-to-Ser exchange at position 55 in strain UVM4 and the Pro-to-Leu exchange at position 222 in strain UVM11 both affect highly conserved residues in the catalytic domain (Fig. 3b). Structural alignment of the Sir2 domains of the wild-type SRTA and the mutated versions from UVM4 and UVM11 based on the 3D structure of the Sir2 domain of human SIRT6 (Supplementary Fig. 3) supports the predicted effect on the catalytic activity in the mutant strains. The mutation in UVM4 likely interferes with the binding of the cofactor NAD(+), while the UVM11 mutation is more likely to affect protein structure and/or the interaction with other proteins.

The *Chlamydomonas* genome contains 17 genes that are annotated as histone deacetylases (HDAC) and three of these are predicted to be of the Sir2-type (subsequently referred to as SRTA encoded by Cre10.g462200/*UVM*, SRTB encoded by Cre12.g524650 and SRTC encoded by Cre05.g234663; Supplementary Table 6). All other histone deacetylases (HDA1-HDA14) are homologous to the *Arabidopsis* histone deacetylases of the RPD3/HDA1 type^24^. Interestingly, *Chlamydomonas* does not possess any member of the HD2-family (type-2 HDACs), which is a plant-specific HDAC family^25^. A phylogenetic tree of all *Chlamydomonas* Sir2-type histone deacetylases and sirtuins from representative model organisms revealed that SRTA is more closely related to the sirtuin-type HDAC from *Arabidopsis* and humans than to SRTB and SRTC (Fig. 3c). In contrast to the *Chlamydomonas* histone deacetylases SRTB and SRTC, which group with the sequence-related phylogenetic sirtuin classes II and III, respectively, the *Chlamydomonas* histone deacetylase SRTA falls into class IV, a class that does not contain prokaryotic members (Fig. 3c; ref. ^26^). Class IV includes the human sirtuins SIRT6 and SIRT7 that are both nucleus-localized and play an important role in epigenetic regulation^27,28^. They catalyze histone deacetylation, but also act on various other cellular substrates, including DNA repair proteins, tumor necrosis factor alpha and a subunit of RNA polymerase I (refs. ^29–31^).

### Introduction of the *SRTA* wild-type allele restores poor transgene expression capacity

**Fig. 4:**
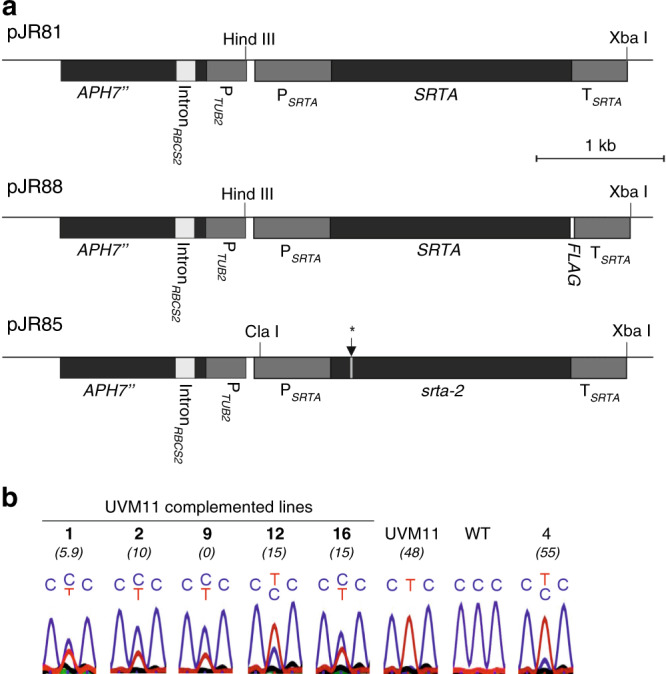
Complementation of the *uvm* mutants with the wild-type SRTA allele. **a** Physical map of the transformation vectors used for complementation of strains UVM4 and UVM11. Exons are shown in black, introns in light gray, and promoter and terminator sequences in dark gray. Expression of the *SRTA* cDNA is driven by the native *SRTA* promoter (P_*SRTA*_), 5’UTR, and 3′UTR (T_*SRTA*_). The hygromycin resistance gene *APH7”* under control of the *TUB2* promoter was used as a selectable marker. Vector pJR88 additionally contains a *FLAG*-tag sequence fused to the *SRTA* coding region. pJR85 served as a control and carries the mutated *srta-2* allele as found in strain UVM4 (location of the mutation indicated by the vertical arrow). **b** The complementation efficiency correlates with the expression strength of the transgenically introduced *SRTA* wild-type allele. Five independent *SRTA* transformants (pJR88) of strain UVM11 were assayed for expression of the endogenous *srta-1* mutant allele relative to the *SRTA* wild-type allele introduced by the transformation. For each complemented line and control strain, the YFP transgene expression capacity is shown in parentheses (YET given in %; cf. Table 1). The cDNA region harboring the *srta-1* point mutation was amplified and sequenced. The height of the T vs. C peaks at the mutated position (nucleotide in the middle and indicated by two letters, with the top one denoting the dominant transcript) is a proxy of relative mRNA abundances. cDNA of UVM11 and the wild type-like strain Elow47 (WT) served as controls. Transformant number 4 that showed only very low *SRTA* transgene expression and, consequently, no complementation (YET = 55%) was included as negative control.

To test whether transgene silencing by the HDAC SRTA is genetically dominant over active transgene expression conferred by the *srta* mutant alleles of strain UVM11 (hereinafter referred to as *srta-1*) and strain UVM4 (hereinafter referred to as *srta-2*) and to confirm our assumption that *srta-1* and *srta-2* represent loss-of-function alleles, complementation analyses were undertaken. To this end, the wild-type cDNA sequence of *SRTA* and that of the *srta-2* mutant allele were cloned in an expression cassette containing the native *SRTA* regulatory sequences (Fig. 4a). Both expression strains were then transformed with these constructs and, additionally, with a FLAG-tagged version of *SRTA* (Fig. 4a; Table 1). Transgenic clones were selected on hygromycin and the presence of the complete *SRTA* cassette was assessed by PCR (for all pJR81 transformants with primers X2-fw and M13uni; Supplementary Table 10). Subsequently, the transgenic strains were supertransformed with the *YFP* reporter to determine their transgene expression phenotype. (The term supertransformation refers to an already transgenic strain that is transformed with another construct.) Supertransformants were selected on paromomycin and, for each *SRTA* transformant, ≥20 supertransformants were analyzed by fluorescence microscopy. While the transformation of UVM4 and UVM11 produced YFP-expressing clones at high frequency (Supplementary Fig. 5), strains that had been transformed with the complementation constructs (pJR81 and pJR88; Fig. 4), produced much lower numbers of YFP-expressing clones (YET: YFP-expressing transformants; as in our mapping strategy, YET values of ≥30% were scored as transgene expression phenotype; Table 1; Supplementary Figs. 4 and 5). Importantly, the complementation efficiency correlated with the expression strength of the transgenically introduced *SRTA* wild-type allele, in that the complemented clones displaying a strong expression of the wild-type allele entirely or nearly entirely lost their transgene expression capacity (Fig. 4b). Complementation was also achieved with the FLAG-tagged *SRTA* sequence (Fig. 4a; Table 1), indicating that the C-terminal tag is compatible with protein function. Quantitation of YFP fluorescence in randomly selected *YFP* supertransformants confirmed the loss of YFP expression capacity in the complemented strains (Supplementary Fig. 5). As an additional control, the UVM4 and UVM11 strains were transformed with the mutated allele *srta-2* (pJR85; Fig. 4a). As expected, this did not result in complementation of the expression phenotype of the strains, as revealed by supertransformation with the *YFP* cassette (Supplementary Table 7).

**Table 1:** Introduction of the *SRTA* wild-type allele into UVM4 and UVM11 restores the poor transgene expression phenotype of the wild type.

Plasmid; strain	YET < 30	YET = 0	YET = 1–10	YET = 11–20	YET = 21–29
pJR81; UVM4	69.2	11.5	34.6	7.7	15.4
pJR81; UVM11	29.2	0.0	20.8	4.2	4.2
pJR88; UVM4	38.9	5.6	5.6	16.7	11.1
pJR88; UVM11	27.8	5.6	11.1	11.1	0.0

Transformants selected on hygromycin were analyzed for their gene expression capacity by supertransformation with the *YFP* reporter gene and analysis of ≥20 supertransformants per line for YFP fluorescence. Complementation was assumed if <30% of the supertransformants showed strong YFP expression. The transformants were grouped according to their transgene expression phenotype as YET (YFP Expressing Transformants) in percent (i.e., number of YFP-expressing transformants relative to the total number of transformants analyzed for each *SRTA*-transformed line). The first column gives the total complementation efficiency (YET < 30%), the second column provides a breakdown into four complementation classes (YET = 0%, YET = 1–10%, YET = 11–20% and YET = 21–29%). Note that, in *Chlamydomonas*, the complementation efficiency is usually lower than 50%, with a major reason being that typically, approximately half of the transformants have not integrated the complete expression cassette into their genome^15^. For further details, see Supplementary Fig. 4.

Finally, we also obtained an insertion mutant (LMJ.RY0402.148523) from the *Chlamydomonas* Library Project (CLiP^32^) that carries a predicted insertion in the *SRTA* locus. The mutant, hereafter named *srta-3*, turned out to harbor a rearrangement involving two loci: Cre10.g462200 on chromosome 10 (encoding SRTA) and Cre03.g200350 on chromosome 3 (encoding SMM12, a putative demethylmenaquinone methyltransferase), with the 5′ junction being in *SRTA* and the 3′ junction in *SMM12* (Supplementary Figs. 6-8; Supplementary Table 8). Due to this potential complication, an insertion mutant in *SMM12* was additionally analyzed (LMJ.RY0402.153656; ref. ^32^; Supplementary Fig. 9). The *srta-3* mutant does not accumulate any *SRTA* transcript (Supplementary Fig. 6b) and, therefore, represents an *SRTA* null allele. Determination of transgene expression efficiency by transformation with the *YFP* reporter gene confirmed that loss of *SRTA* function results in the transgene expression phenotype (Supplementary Table 8). The *smm12* mutant strains did not exhibit detectable YFP fluorescence, as expected.

### Low sensitivity to the silencing of transgenes pre-integrated into the genome

The specific action of SRTA on transgenes raises the question how the enzyme specifically recognizes exogenous DNA sequences. A time point when transgenic DNA could be distinguished from endogenous DNA is prior to or early during the integration of the foreign DNA into the genome. This is because the foreign DNA enters the cell as naked DNA and, possibly, because the DNA repair machinery is recruited to the site of integration. It seems conceivable that the SRTA enzyme specifically acts on chromatin that is assembled de novo, while established chromatin structures are largely protected from its activity.

**Fig. 5:**
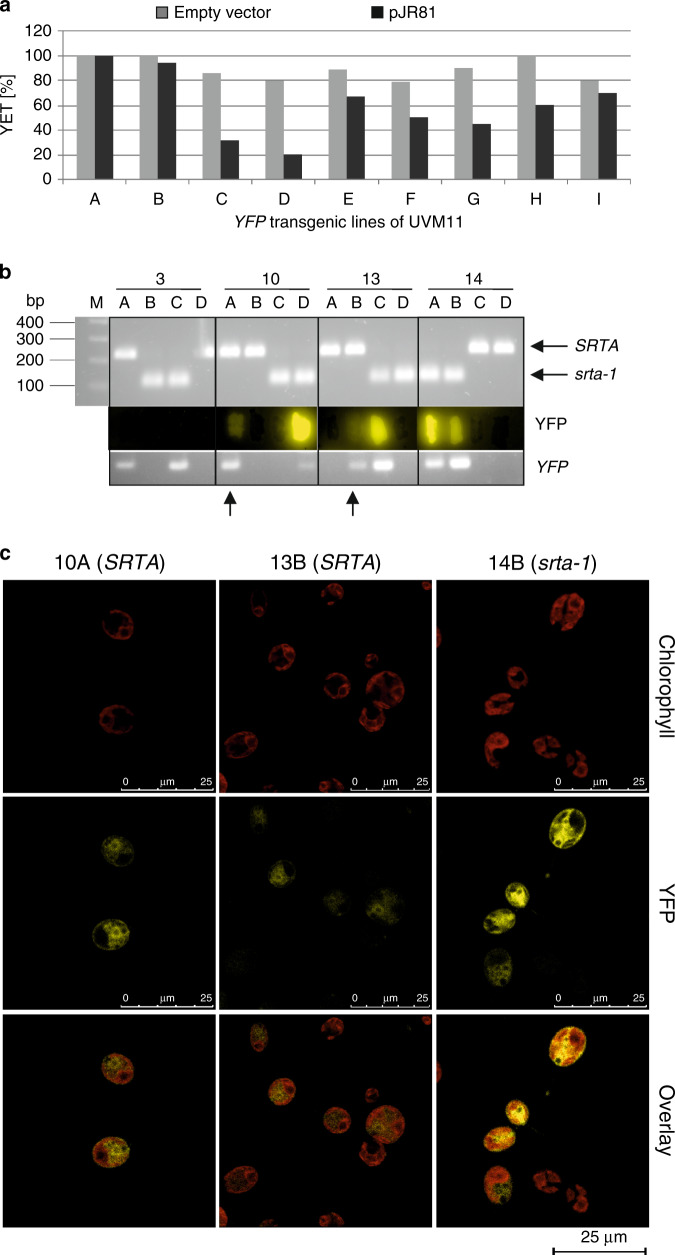
SRTA preferentially silences incoming naked DNA. **a** Complementation of *srta-1* in YFP-expressing UVM11 strains by supertransformation with the *SRTA* wild-type allele. Several independent *YFP* transformants of UVM11 that showed strong YFP fluorescence were supertransformed with plasmid pJR81 (carrying the *SRTA* wild-type allele) and, on average, 20 supertransformants per strain were analyzed for YFP expression. As a control, the same number of supertransformants was generated with the empty vector and analyzed for YFP expression. **b** Epigenetic stability of *YFP* gene expression in meiosis. An expression strain carrying the *srta-1* allele and expressing YFP to high levels was crossed to the wild-type strain CC-1690. Segregants of four tetrads were genotyped for the presence of the wild-type *SRTA* or the mutant (*srta-1)* allele by PCR with primers amplifying a fragment containing the mutation followed by digestion of the PCR amplicon with the restriction enzyme BauI, for which the point mutation in the *srta-1* allele generates a new recognition site. Segregants were also genotyped for the *YFP* cassette, and YFP fluorescence was measured by scanning colonies grown on solid medium. Arrows mark segregants that carry the *SRTA* wild-type allele but nonetheless retain YFP fluorescence. **c** Confirmation of retention of YFP expression in the presence of the *SRTA* wild type-allele by confocal microscopy. Descendants of the crosses shown in (**b**) were analyzed and their genotype is given in parentheses. Results shown in (**b**) and (**c**) were obtained from a single crossing experiment, and similar results were obtained from crosses performed for the mapping analysis of *uvm* using strains UVM11-CW and CC-2290 (alias S1-D2). Source data underlying Fig. 5b are provided as a Source Data file.

To test this hypothesis, we isolated several independent *YFP* transformants of strain UVM11 that displayed strong YFP expression^14,15^, supertransformed them with the *SRTA* wild-type allele (or the empty vector as a control) and measured YFP fluorescence of several supertransformants. While there was a reduction of YFP fluorescence in some of the *SRTA* supertransformants (e.g., strains C, D and G in Fig. 5a), several transgenic strains displayed no detectable loss of YFP fluorescence in the supertransformants (e.g., strains A, B, and I in Fig. 5a).

From these data, we conclude that, once the transgene is stably integrated into the genome and a permissive chromatin state has been established, its expression status can become refractory to the action of the SRTA histone deacetylase. The degree of this insusceptibility may depend on the integration site of the transgene in the genome (Fig. 5a). To also test for meiotic stability of the loss of epigenetic transgene inactivation, we performed crosses of a *YFP*-carrying expression strain with a wild-type strain. After tetrad dissection, individual segregants were tested for YFP fluorescence and genotyped by PCR for the presence of the *srta-1* point mutation and the *YFP* cassette (Fig. 5b, c). Consistent with our earlier observation that YFP expression cannot be used as a marker for mapping (see above), we observed a number of segregants that carried the *SRTA* wild-type allele, but yet expressed YFP (Fig. 5b, c). Thus, even in the absence of the *srta-1* mutation, the *YFP* expression cassette can stay active. This was surprising, given that transgenes usually undergo very efficient silencing upon transformation of wild-type strains.

### Treatments with histone deacetylase inhibitors do not stimulate transgene expression in the wild type

Having identified a mutated histone deacetylase gene as the cause of the superior transgene expression capacity of the UVM4 and UVM11 strains, we next wanted to test if YFP transgene expression can be stimulated in wild-type strains by pharmacological inhibition of HDAC activity. To this end, we exposed *YFP* transformants of the wild type-like strain Elow47 before or after transformation to three different chemicals that are known to act as inhibitors of HDACs.

Application of the HDAC inhibitor trichostatin to *YFP* transformants of Elow47 and UVM11 led to an increase in total levels of acetylated histone 3 after 20 h of incubation with 200 ng mL^−1^ trichostatin (Supplementary Fig. 10), indicating that the drug is taken up and exerts its expected action. Despite the increase in H3ac levels, the treatment did not result in enhanced accumulation of YFP, neither in the Elow47 transformants (where the *YFP* transgenes remained silenced) nor in the transformant of the expression strain (where the YFP expression level remained unchanged). This result is in line with the fact that trichostatin does not inhibit HDACs of the sirtuin class^33^.

Sirtinol and related compounds have been described as potent inhibitors of HDACs of the sirtuin type^33,34^. When cultures of *YFP* transformants of strains Elow47 and UVM4 were incubated with different concentrations of sirtinol for 48 h, none of the treatments resulted in higher levels of YFP accumulation (Supplementary Fig. 11). Also, total levels of acetylated histone H3 remained unchanged (Supplementary Fig. 11).

Finally, we tested the sirtuin inhibitor OSS_128167, which was reported to selectively block activity of SIRT6 in mammals^35^. SIRT6 is the closest human homolog of the *Chlamydomonas* SRTA protein, with both proteins belonging to the class IV of sirtuins (Fig. 3c). Treatment of algal cultures with OSS_128167 did not result in enhanced expression of the *YFP* transgene in three independent *YFP* transformants of Elow47 (Supplementary Fig. 12). Also, YFP accumulation could not be further boosted in a *YFP* transformant of UVM4 (Supplementary Fig. 12), indicating that the chromatin state of the transgene cannot be altered by the drug treatment.

It seems possible that OSS_128167 is not efficiently taken up by algal cells or does not inhibit the *Chlamydomonas* SRTA, although the human SIRT6 is more closely related to the *Chlamydomonas* SRTA than to the human members of sirtuin class I, SIRT1 and SIRT2 (Fig. 3c). Interestingly, SIRT1 and SIRT2 were shown to also be inhibited by OSS_128167, albeit to a much lesser extent (with IC_50_ values that are 10–20 times higher than for SIRT6; ref. ^35^). However, treatment of YFP transformants of Elow47 with increased amounts of the inhibitor (200 µM OSS_128167) also did not show any effect on YFP accumulation. Moreover, immunodetection of acetylated histone H3 versus total H3 revealed no increase in the levels of H3ac after 48 hours of OSS_128167 treatment (Supplementary Fig. 12). Thus, it remains possible that OSS_128167 does not inhibit the *Chlamydomonas* SRTA protein. Alternatively, it could inhibit SRTA, but SRTA affects neither global histone acetylation levels nor the chromatin status of transgenes that have already stably integrated into the genome.

### The transcriptomes of the expression strains, the complemented strains, and their parental strain are very similar

**Fig. 6:**
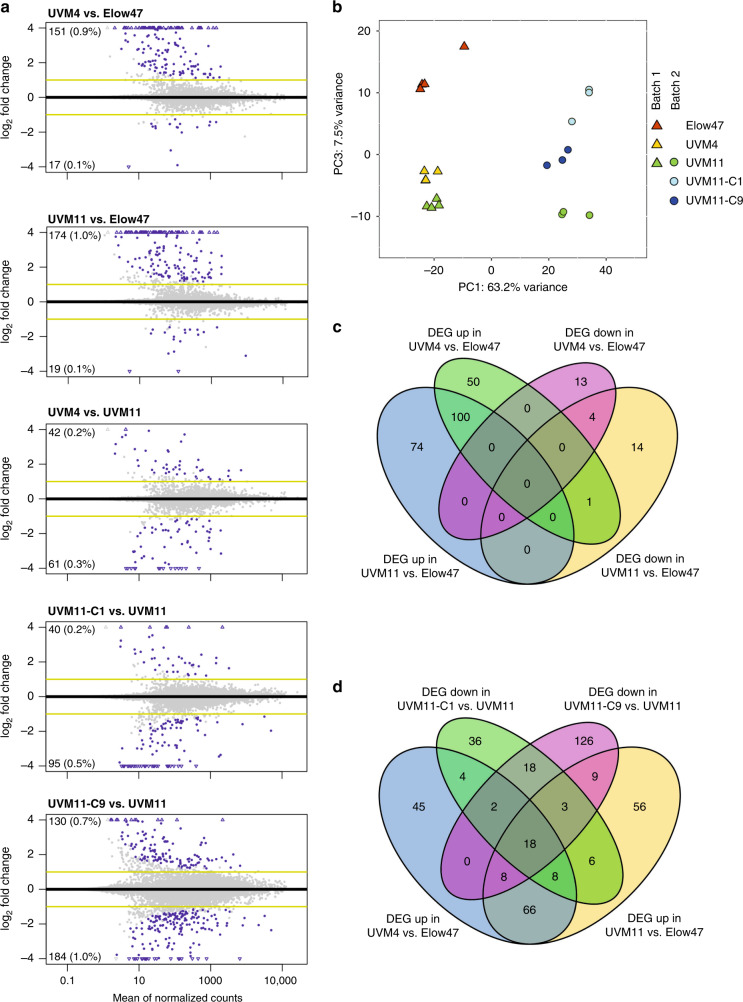
Transcriptome analysis. *srta* mutant strains: UVM4, UVM11; the control strain: Elow47 (harboring the *SRTA* wild-type allele); and two complemented mutants: UVM11-C1 and UVM11-C9. **a** Visualization of differentially expressed genes (DEGs) by MA plot. RNAseq data were analyzed to identify DEGs in head-to-head comparisons between strains as follows: UVM4 (*srta*-2) and UVM11 (*srta*-1) were compared to Elow47 (*SRTA*) and with each other in batch 1. UVM11-C1 and UVM11-C9 (both *SRTA* complemented) were compared to UVM11 (*srta-1*) in batch 2. Log_2_-transformed fold-change values are plotted for all genes. A gene was considered to be a DEG (highlighted in blue) if the absolute value of the log_2_ fold-change was >1 (indicated by an yellow line), and the Benjamini-Hochberg adjusted *p*-value was <0.01. The number of upregulated and downregulated DEGs is indicated in the upper left and lower left corners, respectively, in each panel. **b** Principal component analysis of RNAseq batch 1 consisted of strains Elow47, UVM4 (*srta-2*) and UVM11 (*srta-1*), with four biological replicates each. Batch 2 consisted of strains UVM11 (repeated from batch 1 to identify batch effects), UVM11-C1 and UVM11-C9, with three biological replicates each. A principal component analysis (PCA) plot was constructed from the 1000 genes with the highest variance across all samples. Each replicate is plotted according to the included legend. Note that, if the 1000 genes that are the most variable in this dataset are analyzed, only the third principal component (PC3) corresponds, to a limited extent, with the *SRTA* allele status, and PC3 only represents 7.5% of the variance. This represents a very small effect compared to the differences between batches, even though every effort was made to treat the samples the same. **c** Venn diagram showing the number of DEGs upregulated and downregulated in UVM11 and UVM4 relative to Elow47. Genes were identified as differentially expressed in comparisons between the strains if the fold change was >2, the mean FPKMs across all samples was >1, and the Benjamini-Hochberg adjusted *p*-value was <0.01. **d** Venn diagram of overlap between DEGs as identified from RNAseq analyses of batch 1 and batch 2. In order to identify patterns of expression that potentially correlate with *SRTA* allele status, a Venn diagram of DEGs was generated to identify the overlap between the following four comparisons: the number of upregulated DEGs in UVM11 versus Elow47, the number of upregulated DEGs in UVM4 versus Elow47, the number of downregulated DEGs in UVM11-C1 versus UVM11, and the number of downregulated DEGs in UVM11-C9 versus UVM11. The resulting genes and their expression estimates are presented in Supplementary Data 6 and 7. Source data are provided as a Source Data file.

To distinguish between a general role in transcriptional regulation and a possible transgene-specific action of the SRTA protein, we sought to identify endogenous genes that are affected by the *srta* mutations. To this end, the transcriptomes of both mutant strains (UVM4 and UVM11) were characterized and compared to the transcriptome of their parental strain, the wild type-like control strain Elow47 (RNAseq experiment batch 1). To better filter out differentially expressed genes (DEGs) that are caused by the UV light-induced background mutations in the genomes of UVM4 and UVM11, a second transcriptome analysis was performed in which UVM11 and two independently generated complemented strains of UVM11 were analyzed (RNAseq experiment batch 2). When the datasets from both RNAseq experiments were compared, only a very small percentage of the genes were found to be differentially upregulated or downregulated, with no more than 1.7% of the genes in any given strain showing differential expression (Fig. 6a; Supplementary Data 6 and 7). When a principal component analysis (PCA) on the 1000 genes that are the most variable from the dataset composed of batch 1 and batch 2 was performed, the by far largest principal component (PC1) identifies batch effects (although every effort was made to treat the two sets of samples identically). Only the third principal component (PC3), representing only 7.5% of the variance, corresponds to some extent with the *SRTA* allele status of the strains (Fig. 6b).

Interestingly, strains UVM4 and UVM11 showed only 168 and 193 DEGs in comparison to the control strain Elow47 (Fig. 6a,c). Of those, 151 and 174 genes were upregulated in UVM4 and UVM11, respectively, whereas 17 and 19 genes were downregulated. Strains UVM4 and UVM11 share 100 DEGs that are upregulated and 4 DEGs that are downregulated (Fig. 6c).

If there were any endogenous *Chlamydomonas* genes that are regulated by SRTA, then these genes should be among the class of upregulated genes in UVM4 and UVM11 as identified from our batch 1 RNAseq analysis and, additionally, should be among the class of downregulated genes in the complemented UVM11 strains compared to UVM11 as identified from our batch 2 RNAseq analysis. Remarkably, only 18 genes belong to this category (Fig. 6d; Supplementary Data 6 and 7). Among the 18 shared DEGs, only four genes had an annotation of gene function given and, therefore, no enrichment of specific classes of genes or gene functions could be observed. In addition, those gene candidates generally show rather low expression levels, with the majority having values of 1 FPKM or lower in most samples (Supplementary Data 6 and 7). Although it currently cannot be excluded that some of these genes are deregulated due to the loss of SRTA, their very low number (compared to the total number of protein-coding genes in the genome of *Chlamydomonas* which is more than 17,700; ref. ^36^) excludes a general role of SRTA in chromatin modification and transcriptional regulation.

Interestingly, the transcript abundance of *SRTA* is also unchanged in the mutant strains compared to the control strain (Supplementary Fig. 13), suggesting that the mutations in strains UVM4 and UVM11 do not affect mRNA half-life and, moreover, indicating that there is no compensation mechanism to the SRTA deficiency that would operate at the level of transcription or mRNA stability.

Prior to RNAseq analysis, both complemented strains (UVM11-pJR88-1 and UVM11-pJR88-9; cf. Fig. 4b), which had been generated four years ago and maintained on hygromycin-containing medium ever since, were re-analyzed for their complementation efficiency. Both strains still showed unaltered strong expression of the introduced *SRTA* wild-type allele, as determined from the ratio of sequenced *SRTA* versus *srta* cDNA amplicons as well as from the RNAseq data. A new supertransformation of the strains with the *YFP* cassette confirmed the functional complementation in that most of the supertransformed colonies showed no (or only very weak) YFP fluorescence. This result shows that the wild-type form of the SRTA enzyme does not repress its own expression by acting on the (transgenically introduced) *SRTA* genomic locus. Instead, normal-level *SRTA* expression is maintained and the SRTA enzyme acts only on newly introduced transgenic DNA, as evidenced by supertransformation with the *YFP* cassette. This finding lends further support to our hypothesis that, once the chromatin status of the transgene is established (prior to or upon integration into the genome), it remains remarkably stable over time.

In sum, the very small changes in the transcriptomes and the lack of evidence for specific effects of the mutations in *SRTA* on endogenous gene expression argue against a general role of SRTA in the regulation of nuclear gene expression in *Chlamydomonas*. These results are in line with our previous findings that, apart from the strong expression of foreign genes, there is no other detectable phenotype of the expression strains^15^. Taken together, these results raise the interesting possibility that the SRTA protein acts mainly on naked DNA that enters the cell (i.e., transgenic DNA and possibly other types of invading DNA sequences), and inactivates them through the establishment of a repressive chromatin structure.

### Genome-wide analysis of transposon mobility

A major function of silencing pathways is to control mobile genetic elements^37,38^. Our DNA gel blot analyses (Fig. 1f) had provided preliminary evidence for suppression of transposon mobility not being affected in our expression strains. Having obtained whole-genome sequencing data for the mutant strains UVM4 and UVM11 as well as the control strains Elow47 and cw15arg- enabled us to comprehensively assess the effect of the *SRTA* mutations on transposon activity in the expression strains (Supplementary Data 8). To this end, the DNA sequencing datasets were used to perform a genome-wide survey of nine different transposons in the four strains. In total, 656 transposons were found, and the vast majority of them (96%) were in the same loci in all four strains (Table 2). The retrotransposon MRC1 was somewhat exceptional in that it showed multiple transposition events, which however, were not correlated to the presence of an *SRTA* mutation. Instead, the presence of multiple new insertions in all strains (including the control strains) suggests that the MRC1 transposon is generally more active than the other transposons investigated.

**Table 2:** Presence of transposons in the wild-type strain cw15arg-, the wild type-like strain Elow47 and the *srta* mutant strains UVM4 and UVM11 as determined by whole-genome sequencing (cf. Supplementary Data 8).

Transposon	Total number	Present in all strains	In cw15arg- only	In Elow47 only	In UVM4 only	In UVM11 only	In Elow47 + UVM4 + UVM11
MRC1	178	154	3	3	4	7	7
Bill	12	12	0	0	0	0	0
Gulliver	274	273	0	0	0	0	1
Pioneer1	2	2	0	0	0	0	0
REM1	12	12	0	0	0	0	0
Tcr1	8	8	0	0	0	0	0
Tcr3	59	57	0	1	0	1	0
TOC1	59	59	0	0	0	0	0
TOC2	52	52	0	0	0	0	0

From these data, it can be concluded that the mutant strains do not show enhanced mobility of transposable elements, and that SRTA is unlikely to play a substantial role in suppression of transposon activity in *Chlamydomonas*.

## Discussion

In this study, we have identified the molecular basis of the strong transgene suppression in the reference alga *Chlamydomonas*. Performing mapping and genotyping by transformation, we identified the expression-conferring mutations in a highly conserved domain of a Sir2-type histone deacetylase (Cre10.g462200 encoding SRTA). Sirtuins represent a highly conserved family of NAD(+) dependent protein deacetylases with the yeast histone deacetylase Sir2 (silent information regulator) as the founding family member^39^. The algal expression strains UVM4 and UVM11 both carry non-synonymous point mutations in the *SRTA* gene, at different positions within the highly conserved Sir2 domain. Complementation experiments and characterization of an insertion mutant line confirmed the functional role of the NAD(+) dependent HDAC in epigenetic transgene suppression.

Knock-out of *SIRT6*, a representative of the same sirtuin clade (class IV; Fig. 3c; ref. ^26^) and the closest homologue of SRTA in mammals, causes a severe phenotype in mice. This is unsurprising given that SIRT6 binds to the promoter region of thousands of genes in mice fibroblasts^40^. By contrast, the *Chlamydomonas* SRTA acts very differently from its mammalian SIRT6 homolog in that *srta* mutants have no discernable phenotype^15^. Moreover, the results of our comparative RNAseq analyses (Fig. 6) exclude a function of SRTA as a general transcriptional regulator of endogenous genes in the genome.

Instead, our results indicate that the effect of the loss of SRTA function is largely transgene-specific. Endogenous genes remain unaffected, as evidenced by our comparative transcriptomic analysis, suggesting that SRTA may act preferentially on DNA that newly enters the cell, is naked and not yet covered by chromatin proteins. Once the foreign gene is integrated into the genome and an active expression status has been established, it is much less susceptible to SRTA-mediated silencing than upon initial introduction into the cell. It should be noted, however, that the action of SRTA is not entirely limited to unintegrated naked DNA. In fact, the genetic screen that led to the isolation of the UVM4 and UVM11 mutants was based on the post-integration loss of transgene silencing^15^, suggesting that, at least under certain conditions, loss of SRTA function can also lead to activation of silent transgenes. The UV mutagenesis applied in the genetic screen could be one such condition in that it may have triggered DNA repair pathways that involve transient removal of histones.

An alternative explanation for the high transgene expression capacity of the *srta* mutants would be that the loss of SRTA predisposes the transgenes to insert into more favorable (euchromatic) genomic locations for expression. Once a transgene has been inserted into such a location, its continued expression may no longer depend on the presence of the *srta* mutation. In view of our findings that SRTA function is not involved in the control of endogenous gene expression (Fig. 6) and its loss does not lead to global changes in chromatin structure (Fig. 1; Supplementary Fig. 2), we consider this explanation less likely. Since the mutations identified in the two expression strains affect the catalytic domain of SRTA (and gene expression levels are unaffected), the phenotype is most likely associated with HDAC activity and the setting of histone marks on transgenic DNA entering the nucleus. Also, our ChIP data suggest that, in the expression strains, transgenes can also integrate into chromosomal regions that carry heterochromatic marks (Fig. 1e).

Genome resequencing and a genome-wide survey of the copy numbers and locations of transposable elements have allowed us to also exclude a role of SRTA in the control of mobile genetic elements. This, together with the unaltered sensitivity of the mutant strains to chemically induced DNA damage, the absence of significant effects of SRTA on the expression of endogenous genes in the genome and the lack of any detectable phenotype of *srta* mutants, suggests that SRTA plays no substantial role in the maintenance of genome stability or in nuclear gene regulation. Instead, all this evidence points to a transgene-specific action of SRTA in that it specifically silences foreign DNA after its entry into the cell, perhaps even prior to (or concurrently with) its integration into the genome. The inability of several pharmacological inhibitors of HDACs to phenocopy the expression phenotype of *srta* mutants may lend further support to this hypothesis. We currently cannot exclude the possibility that the primary recognition of the foreign DNA is mediated by an unknown protein factor that then recruits SRTA to assemble a repressive chromatin state. Nonetheless, even if additional factor(s) are involved in the molecular recognition process, SRTA appears to be the crucial factor that initiates silencing in a transgene-specific manner.

Invading nucleic acids are particularly prevalent in aquatic environments and, due to the absence of a protected germline, unicellular organisms are particularly susceptible to foreign nucleic acid sequences. We, therefore, speculate that SRTA silencing of foreign DNA may have evolved as a protective mechanism to prevent the expression of viruses and other potentially deleterious types of environmental DNA sequences that may constantly enter algal cells through horizontal DNA transfer^41^. Interestingly, only relatively few lytic viruses are known in eukaryotic algae, and not a single virus is known that would infect *Chlamydomonas*^42,43^, possibly suggesting that algae have evolved unusually strong protective mechanisms against infectious nucleic acids.

In summary, we conclude from our data that foreign DNA that enters the cell is the preferred target of SRTA-mediated silencing. This suggests that SRTA may provide protection from potentially harmful types of environmental DNA. Further work will be needed to test, if naked DNA (that is not yet covered by chromatin proteins) is the only target of SRTA-mediated silencing, and if *srta* mutants have a selective disadvantage under conditions that are linked to the exposure to invading environmental DNA, including DNA viruses that infect other species of green algae.

Finally, the absence of any substantial deregulation of the transcriptome upon mutational inactivation of *SRTA*, the lack of evidence for genome destabilization by unleashed transposons, and the long-term stability of high-level transgene expression make the UVM strains a highly valuable tool for the efficient expression of foreign genes in *Chlamydomonas*.

## Methods

### Algal strains and cultivation conditions

*Chlamydomonas reinhardtii* strains Elow47, UVM4 and UVM11 were generated in a previous study^15^. Strain Elow47 is a co-transformant of the arginine auxotrophic strain CC-4350 (*cw15*, *mt*+, *arg7;* alias *cw15arg-*) that carries the *CRY1-1* emetine resistance gene and the *ARG7* gene providing arginine prototrophy. The expression strains UVM4 and UVM11 were obtained by UV-light-induced mutagenesis of strain Elow47, followed by selection for high transgene expression capacity^15^. *Chlamydomonas* strains CC-1690 (*mt*+; Sager 21gr), CC-124 (*mt−*), and the polymorphic strain CC-2290 (*mt−*, alias S1-D2) were obtained from the *Chlamydomonas* Stock Center (http://chlamycollection.org/strains/). The insertion mutants *srta-3* (LMJ.RY0402.148523, Cre10.g462200) and *smm12* (LMJ.RY0402.153656, Cre03.g200350) and the corresponding wild-type strain CC-5325 are from the *Chlamydomonas* mutant library (CLiP, https://www.chlamylibrary.org/) and were provided by the *Chlamydomonas* Stock Center.

Cells were grown photomixotrophically in liquid Tris-acetate-phosphate (TAP) medium^44,45^ on a rotary shaker at 22–25 °C or on agar-solidified TAP medium under continuous light (50–100 µE  m^−2^ s^−1^, cool-white fluorescent lamps). Arginine (100 µg mL^−1^) was added to the medium as required. Cell numbers were determined by counting of 100-fold diluted samples in a Z2 Coulter Counter (Beckman Coulter, Krefeld, Germany).

### Cloning of *SRTA*, *srta-2* and construction of transformation vectors

Cloning of the coding sequence, the promoter/5′UTR and the 3′UTR sequence of *SRTA* (Cre10.g462200) was performed on the basis of the gene model provided by Phytozome v10 (http://www.phytozome.net/). The sequence comprising the reading frame of the mutated *srta-2* allele (carrying the point mutation detected in strain UVM4) and additional 31 bp upstream was amplified with primers SRT3402fw (5′-TACTAGCTTACGAACAGCGCC-3′) and SRT3402rvEcoRI (5′-agaatTCACGCGTCATCGTCACC-3′) using oligo(dT)-primed cDNA of strain UVM4 as a template and introducing an EcoRI site (underlined) at the 3′ end of the amplicon, followed by cloning into the pCR^TM^ 2.1-TOPO® TA vector (Invitrogen, Karlsruhe, Germany). To clone the promoter of *SRTA*, the 5′ end of the gene was amplified with primers P-SRT-BamHI-fw (5′-aggatCCGCGCCATGTCCC-3′, nucleotides introducing a BamHI site underlined) and NheI-SRT-rv (5′-CGCTAGCTCCTTGACGCCC-3′, NheI site underlined) using genomic DNA of strain Elow47 as a template. The 3′UTR of *SRTA* was amplified from the same template using primers T-SRT-EcoRIfw (5′-agaattcAGGTGCTGTGTTGGGATG-3′, nucleotides introducing an EcoRI site underlined) and T-SRT-XbaIrv (5′-TTCTAGACGTCGATGTATCCTGTGGTG-3′, XbaI site underlined), and cloned into the pCR^TM^ 2.1-TOPO® TA vector (Invitrogen). Subsequently, the *srta-2* coding sequence-containing TOPO vector was digested with BamHI and NheI and ligated to the similarly digested PCR product of the 5’ end of the *SRTA* gene. The resulting pTOPO-P-uvm4 vector was digested with EcoRI and XbaI, and ligated to the insert of the similarly digested pTOPO vector that contained the *SRTA* 3’UTR sequence, resulting in plasmid pTOPO-P-uvm4-T.

Plasmid pJR80 was constructed by digesting plasmid pTOPO-P-uvm4-T with BamHI and XmaI, and replacing the 5′ end of the *srta-2* sequence containing the point mutation with the similarly digested *SRTA* wild-type sequence. The *SRTA* sequence was amplified by an overlap extension PCR in which amplicon 1 (generated with primers P-SRT-BamHI-fw and SRT-E2wtfw [5′-GTGTTCACGGGCGCC**G**GCATCTCCACCGCCTGCGGCATCC-3′, nucleotide reverting the point mutation in *srta-2* in bold] and using pTopo-P-uvm4-T as template) and amplicon 2 (generated with primers SRT-E2wt-rv [5′-GGATGCCGCAGGCGGTGGAGATGC**C**GGCGCCCGTGAACAC-3′, nucleotide reverting the point mutation in *srta-2* in bold] and SRTXmaI-rv [5′-ACCCCGGGGCCACTGTCAG-3′] using pTOPO-P-uvm4-T as template) were used as templates and amplified with primers P-SRT-BamHI-fw and SRTXmaI-rv.

*Chlamydomonas* transformation vector pJR85 was obtained by digestion of plasmid pJR43 (a derivative of the *APH7”*-containing pHyg3 vector; ref. ^46^) with XbaI and HindIII followed by ligation to the 2988 bp, gel-eluted XbaI/HindIII fragment of pTOPO-P-uvm4-T that contained the *srta-2* sequence including the *SRTA* promoter and terminator sequences. Plasmid pJR43 was generated by ligating the purified KpnI/EcoRV hygromycin cassette (*P*_*TUB2*_::*APH7”*) from pHyg3 into the similarly digested pBC KS(+) vector.

*Chlamydomonas* transformation vector pJR81 carrying the *SRTA* sequence under the control of the *SRTA* promoter and terminator sequences was obtained by digesting plasmid pJR85 with ClaI and SmaI, and ligating the eluted vector with the gel-eluted insert from the similarly digested plasmid pJR80. Plasmid pJR88 was obtained by MluI digestion of plasmid pJR81 followed by dephosphorylation using Fast Alkaline Phosphatase (Thermo Fisher Scientific, Darmstadt, Germany) and ligation to the annealed double-stranded oligonucleotide sequence that has MluI-compatible single-stranded overhangs at the 5′ and 3′ ends, and encodes the FLAG sequence. The dsDNA fragment was generated by annealing sense (SRT-FLAGfw, 5′ cgcgGACTACAAGGACGACGACGACAAGTAA 3′) and antisense oligonucleotide (SRT-FLAGrv, 5′ cgcgTTACTTGTCGTCGTCGTCCTTGTAGTC 3′) followed by phosphorylation with T4 polynucleotide kinase (New England Biolabs, Frankfurt/M., Germany).

In addition to the Venus *YFP*-containing vector pJR39 (ref. ^15^) and vector pRMB12 containing the fully codon-optimized *CrYFP* ^14^ that have the *APHVIII* gene as a selectable marker, another Venus *YFP*-containing plasmid with a different selectable marker gene was constructed (pJR91 carrying the *APH7”* gene). pJR91 was obtained by digestion of pJR43 and isolation of the *APH7”*-containing EcoRV/KpnI restriction fragment, followed by ligation to vector pJR39 cut with XhoI followed by treatment with the Klenow fragment of DNA polymerase I (New England Biolabs, Frankfurt/M., Germany) and digestion with KpnI.

### Transformation of *Chlamydomonas reinhardtii*

Nuclear transformation of cell wall-deficient *Chlamydomonas* strains was performed using the glass bead-assisted method^47,48^. Transformations of cell wall-containing strains were done by electroporation using the Eppendorf 2510 electroporator or the Biorad Gene Pulser Xcell^TM^ electroporation system. All transformation vectors were linearized with a restriction enzyme (RE) that has a unique recognition site within the vector backbone. One microgram of linearized plasmid was used for the transformation of 1 × 10^8^ cells harvested from a culture grown until the early to mid-exponential phase. For electroporation, pelleted cells were dissolved in sterile water with 40 mM sucrose in a volume of 500 µL and transferred to a pre-cooled electroporation cuvette (4 mm gap; Biorad, Germany). Cells were incubated with the linearized plasmid DNA for 5–20 min on ice before an electric pulse of 1200 V was applied. After electroshock, cuvettes were immediately cooled on ice and cells were transferred into 11 mL TAP medium with 40 mM sucrose for recovery under non-selective conditions for ~4 h. The cells were then pelleted and spread onto plates with selective medium. Supplementary Table 9 shows an overview of all transformation experiments conducted in this study, including information about the RE used for linearization of the vectors and the conditions used for the selection of transformants.

### DNA isolation and standard PCRs

Extraction of genomic DNA was performed according to published protocols^14^ using 2 mL of algal cultures grown in liquid medium to late-exponential phase or a similar number of cells grown on solid medium. The cell pellet was resuspended in 200 µl extraction buffer (2% cetyltrimethylammonium bromide, 100 mM Tris–HCl pH 8.0, 20 mM EDTA pH 8.0, 1.4 M NaCl, and 2% freshly added β-mercaptoethanol) by vortexing, followed by the addition of 200 µL chloroform/isoamyl alcohol (24:1) and incubation at 65 °C for 20 min under shaking (1400 rpm). After centrifugation at 10 °C for 10 min at maximum speed, the DNA was precipitated by adding 0.7 volume of isopropanol to the supernatant. The pellet was washed with 70% (v/v) ethanol and the air-dried pellet was resuspended in 50 µL water supplemented with 1 µL RNase A (10 mg mL^−1^), and incubated for 15 min at 37 °C.

Alternatively, crude DNA extracts were isolated for PCR genotyping according to the instructions given by the *Chlamydomonas* mutant library (CLiP, https://www.chlamylibrary.org/) with minor modifications. In brief, *Chlamydomonas* cells collected from plates were resuspended in 50 µL of 10 mM EDTA (pH 8.0) by vortexing for 10 s, followed by incubation at 100 °C for 10 min in a thermocycler. After cooling for 1 min at 4 °C, the samples were again vortexed for 10 s and centrifuged for 1 min at 2000 × *g*. The DNA-containing supernatant was then transferred to fresh tubes and used for PCR analysis. Polymerase chain reactions (PCRs) were performed according to standard protocols (1 min at 98 °C, 90 s at 58–66 °C, 90 s at 72 °C; 36–40 cycles) using GoTaq® DNA polymerase (Promega, Mannheim, Germany). Primer sequences are listed in Supplementary Table 10.

### Southern blot analysis to determine transposon copy numbers

Samples of 40 or 60 µg total DNA were digested with the RE HincII or HindIII to analyze the copy number of the retrotransposon-like element *TOC1* and the DNA transposon *Gulliver*, respectively. Digested total DNA samples were separated in 1% agarose gels and transferred onto Hybond^TM^ N^+^ nylon membranes (GE Healthcare) by capillary blotting. The *TOC1* probe was amplified by PCR (primers TOCpos207 5′-GGGACGGTGACCTCAGTGTGTCGC-3′ and TOCpos474 5′-CCGCTCTGCTCCGATTTGCTCCCG-3′), cloned into the pCR^TM^ 2.1-TOPO® TA vector (Invitrogen), digested with EcoRI and purified by agarose gel electrophoresis followed by labeling with ^[α-32P]^dCTP by random priming (Megaprime^TM^ DNA labeling system, GE Healthcare, Freiburg, Germany). The *Gulliver* probe was amplified by PCR (GULLIVfw2 5′-CGCAGGCAAGCGAAGGCTCA-3′ and LTRrev 5′- AAGTGCCTTTGTGGAAGCCATGT-3′) and purified by gel electrophoresis prior to labeling. Hybridizations took place at 65 °C using standard protocols.

### Protein isolation and immunoblot analysis

Isolation of total soluble protein extracts, separation of proteins via SDS page and immunoblot analysis were performed according to published protocols^15^ with minor modifications. In brief, algal cell pellets were resuspended in 400 µL lysis buffer [50 mM HEPES/KOH pH 7.5, 10 mM KAc, 5 mM MgAc, 1 mM EDTA, 1 mM DTT, 1× protease inhibitor cocktail cOmplete (Roche)], followed by disruption of cells by sonication (Sonifier^®^, W-250 D) at 10% amplitude for 15 s while keeping samples on ice. After centrifugation at 4 °C for 10 min (10,000–12,000 × *g*), the supernatant was transferred to a fresh tube and protein concentration was determined using the Bradford assay (Roti^®^Quant, Roth, Karlsruhe). Heat denaturation of the protein samples in 1× sample buffer took place at 75 °C for 7 min or at 95 °C for 5 min. Protein samples were separated in denaturing 15% SDS-PAA gels and transferred onto PVDF (polyvinylidene difluoride) membranes (Hybond^TM^; GE Healthcare, UK) using standard Tris-glycine transfer buffer (25 mM Tris/HCl, 192 mM glycine, pH 8.3). After electrophoretic transfer, membranes were treated with blocking buffer composed of 0.5% BSA and 0.5% milk powder in 1× TBS-T.

For immunobiochemical protein detection of global histone H3 and specific histone modifications the following primary antibodies were used in different dilutions in TBS-T: 1:50,000 dilution of anti-H3 (ab1791; Abcam, Cambridge, UK), 1:10,000 dilution of anti-H3ac (03-599; Merck, Darmstadt, Germany), 1:1000 of anti-H4ac (K5, 8, 12, 16; AHP418; Biorad), 1:5000 of anti-H3K4me1 (ab8895; Abcam) and 1:1000 of anti-H3K4me2 (07-030; Merck). Detection of YFP was performed with a 1:1000 dilution of monoclonal anti-GFP primary antibody (632381; Clontech, Mountain View, CA). Gels were loaded based on equal amounts of total soluble protein. Immunochemical detection was performed with the ECL Plus^TM^ detection system (GE Healthcare) and a goat anti-rabbit secondary antibody (170-6515; Biorad, Munich, Germany) or, for YFP detection, a goat anti-mouse HRP-conjugated secondary antibody (AS111772; Agrisera, Vännäs, Sweden), using a 1:10,000 dilution.

### Chromatin immunoprecipitation

For each ChIP experiment, at least 1200 *YFP* transformants (with vector pJR39) of each strain (Elow47, UVM4, and UVM11) were pooled and a total of 10^8^ algal cells were harvested by a 2-min centrifugation at 4 °C and 3220 × *g*. The chromatin was cross-linked by resuspending cells in 10 mL of freshly prepared cross-linking buffer (20 mM HEPES-KOH, pH 7.6, 80 mM KCl, 0.35% formaldehyde), and incubated for 10 min at 24 °C. Subsequently, cross-linking was quenched by the addition of glycine to a final concentration of 125 mM, and continued incubation for 5 min at 24 °C. Cells were then pelleted by centrifugation for 2 min at 4 °C and 3220 × *g*, followed by cell lysis by addition of 400 μL lysis buffer (1% SDS, 10 mM EDTA, 50 mM Tris-HCl, pH 8.0, 0.25× protease inhibitor cocktail [Roche]) and sonication on ice using a BANDELIN Sonopuls HD 2070 sonicator with sonication tip MS 73 (55% output control and 60% duty cycle) to obtain an average DNA fragment size of ~200 bp. ChIP was performed according to published procedures^49^. In brief, culture aliquots corresponding to ~2 × 10^7^ algal cells were diluted 1:10 with ChIP buffer (1.1% Triton X-100, 1.2 mM EDTA, 167 mM NaCl, 16.7 mM Tris-HCl, pH 8), and supplemented with BSA and sonicated λ-DNA at final concentrations of 100 and 1 μg mL^−1^, respectively. Antibodies specifically recognizing the following epitopes were used: histone H3 (5 μL; Abcam ab1791); diacetyl H3K9 and H3K14 (10 μL; Upstate 06-599); tetra-acetyl H4K5, H4K8, H4K12, and H4K16 (10 μL; Upstate 06-866); monomethylated H3K9 (5 μL; ab9045; Abcam). Antibody-protein-DNA complexes were incubated for 1 h at 4 °C. Subsequently, 6 mg preswollen protein A Sepharose beads (Sigma-Aldrich) were added, followed by a 2 h incubation at 4 °C. Sepharose beads were pelleted by centrifugation at 4 °C for 30 s at 12,000 × *g*, washed with washing buffer 1 (0.1% SDS, 1% Triton X-100, 2 mM EDTA, pH 8) containing 150 mM NaCl, followed by additional washes with washing buffer 1 containing 500 mM NaCl and washing buffer 2 (250 mM LiCl, 1% Nonidet P-40, 1% Na-deoxycholate, 1 mM EDTA, 10 mM Tris-HCl, pH 8), and two washes with TE (1 mM EDTA and 10 mM Tris-HCl, pH 8). Protein-DNA complexes were then eluted by incubation for 30 min at 65 °C in elution buffer (1% SDS, 0.1 M NaHCO_3_). Cross-links were reverted by overnight incubation at 65 °C after the addition of NaCl to a final concentration of 0.5 M. Proteins were then digested by addition of 3.5 μg mL^−1^ proteinase K, 8 mM EDTA, and 32 mM Tris-HCl, pH 8.0, followed by incubation for 1 h at 55 °C. The solution was subsequently extracted once with phenol/chloroform/isoamyl alcohol (25:24:1) and once with chloroform/isoamyl alcohol (24:1), and the DNA was precipitated by incubation with 2 volumes of ethanol after addition of 0.3 M Na-acetate, pH 5.2, and 10 μg mL^−1^ glycogen for 3 h at −20 °C. Precipitated DNA was pelleted by a 20 min centrifugation at 4 °C and 16,000 × *g*, washed with 70% ethanol, air-dried, and resuspended in TE buffer. Aliquots representing 2.5% of the precipitated DNA were used as templates for qPCR. Signals for individual gene regions were normalized to 10% input DNA and then to the corresponding signal derived from the *CYC6* promoter (H3, AcH3, AcH4) or a TFR (TFR; H3K9me1). qPCR assays were performed using the StepOnePlus RT-PCR system (Applied Biosystems) and the Maxima SYBR Green kit (Fermentas). Each amplification reaction contained the vendor’s master mix, 200 nM of each primer and ChIPed DNA corresponding to 2.5% of the total eluate. The reaction conditions were: 95 °C for 10 min, followed by cycles of 95 °C for 15 s and 65 °C for 60 s, up to a total of 40 cycles. Primers used for qPCR are listed in Supplementary Table 10.

### Crossings, mapping, and PCR marker analysis

Crosses were performed according to standard procedure^44^. Prior to the mapping of the mutated locus in the expression strains, the cell wall-deficient strain UVM11 (*mt*+) that displayed poor mating capability was back-crossed to the mt- wild-type strain CC-124 (Fig. 2a) to restore flagella as a prerequisite for efficient mating. From this cross, the offspring strain UVM11-CW (*mt*+) was isolated that showed strong YFP expression as confirmed by microscopy analysis of several independent *YFP* transformants. Strain UVM11-CW (*mt*+) was then crossed to mapping strain CC-2290 (*mt-*; alias S1-D2) and the progeny was screened for its transgene expression capacity by transforming every single segregant with the *YFP* reporter gene followed by YFP fluorescence analysis. Progeny with high YFP expression was used as the mapping population and selected for PCR marker analysis using the *Chlamydomonas* mapping kit available from the *Chlamydomonas* resource center (http://www.chlamycollection.org/products/mapping-kits/). Newly developed markers for fine mapping are listed in Supplementary Table 10. Segregants were scored as having strong YFP expression capacity (i.e., showing the expression phenotype) if at least 30% of the colonies analyzed showed clearly visible YFP fluorescence. On average, 20 independent transformants were analyzed.

### Zeocin sensitivity assay

To analyze the sensitivity of the mutant strains UVM4 and UVM11 to DNA-damaging agents, zeocin (Invivogen) sensitivity assays were performed. Cells of a late-exponential culture were diluted to three different cell concentrations (1 × 10^7^, 1 × 10^6^, and 2 × 10^5^ cells mL^−1^) and from each dilution, aliquots of 8 µl were dropped onto TAP agar plates containing different concentrations of zeocin (0, 1, 2, and 4 µg mL^−1^). Photos of the drop tests were taken after ten days.

### Analysis of GFP and YFP fluorescence

Fluorescence of YFP was detected in living cells with a fluorescence stereomicroscope (Axioskop 2; Zeiss, Göttingen, Germany) equipped with appropriate filters for YFP (excitation BP 450–490 nm, FT 510, emission BP 515–565 nm, or excitation BP 470/20 nm, FT 493, emission BP 505–565 nm) and chlorophyll fluorescence (excitation BP 450–490 nm, FT 510, long pass filter 520 nm). Alternatively, the detection was done with a confocal laser-scanning microscope (TCS SP5; Leica, Wetzlar; Germany) using an argon laser for excitation (at 514 nm for YFP), a 510–535 nm filter for detection of YFP fluorescence and a 630–720 nm filter for detection of chlorophyll fluorescence. YFP fluorescence intensity was measured with the CLARIOstar® microplate reader (BMG LABTECH GmbH, Ortenberg, Germany) in transparent 96-well plates with cultures of 100 µl *Chlamydomonas* cells per well (grown to mid-exponential phase) using bottom optics and excitation (508 nm, bandwidth 15 nm) and emission (551 nm, bandwidth 20 nm) settings suitable for YFP detection (dichroic 528.2). Scanning of *Chlamydomonas* clones grown on TAP agar plates for YFP fluorescence was conducted with the Typhoon scanner (GE Healthcare) applying the 526 SP Fluorescein filter.

### RNA isolation and cDNA synthesis

Total RNA was isolated from algal cultures grown to late-exponential phase with the Direct-Zol^TM^ RNA MiniPrep kit (Zymo Research, Freiburg, Germany) followed by an additional DNase treatment using TURBO DNase (Thermo Fisher Scientific) according to the manufacturer’s instructions. RNA yield and quality were assessed by determining the 260 nm/280 nm absorbance ratio using a Nanodrop instrument (Thermo Fisher Scientific).

Unless otherwise stated, first-strand cDNA was synthesized from total RNA (1.7–4 µg) using the RT primer mix (Qiagen) and Superscript III reverse transcriptase (Invitrogen) following the manufacturer´s protocol. Subsequently, the RNA was removed by RNase H (Invitrogen) treatment. The absence of contaminating genomic DNA was confirmed by the inclusion of control samples that had not undergone reverse transcription. In order to determine the transcript ratio of the *srta-1* mutant allele and the *SRTA* wild-type allele in the complemented lines of strain UVM11, the *SRTA*-specific primer SRT3402r2 (5′- GCTCGCCGGTCCTTGG-3′) was used to prime the reverse transcription reactions. The sequence containing the *srta-1* point mutation was amplified with primers SRTE5f (5′-AGCTGGTGGACAACATCCTG-3′) and SRT8r (5′-GCGTCTTCTGCAGGTTCAC-3′).

### Whole-genome resequencing

G`enomic sequencing was performed on strains Elow47, UVM4, UVM11, and the wild-type cw15arg- according to published protocols^50^. In brief, 1 µg of genomic DNA was used to prepare sequencing libraries using the TruSeq DNA sample preparation kit, version 1 (Illumina) following the low-throughput protocol. The resulting libraries were sequenced with paired-end 100 + 100 nt reads on a HiSeq2000 sequencer (Illumina). Raw sequencing reads were aligned to version 5 of the *Chlamydomonas* reference sequence from Phytozome (http://phytozome.net) with BWA mem, version 0.7.5a-r405 (ref. ^51^), using default parameters. Duplicate read pairs were removed using Picard MarkDuplicates, version 1.85(1345) (http:// broadinstitute.github.io/picard) with default parameters. Variants were identified with the Genome Analysis Toolkit (GATK), version 2.6-5-gba531bd. Loci that were identified as a reference in Elow47, but variants in either UVM4, UVM11 or both were selected for further analysis. The functional impact of each variant was predicted using SnpEff version 4.3r (ref. ^52^). This produced a table of variants in VCF format, which was manually curated to identify the genes impacted by SNVs and InDels in either UVM4, UVM11, or both.

The sequencing reads are available from the NCBI Sequence Read Archive under accession numbers SRR1797981 (cw15arg-), SRR6872092 (Elow47), SRR6872091 (UVM4), and SRR6872090 (UVM11).

### Transposon analysis

Paired-end (100 + 100 nt) DNAseq was performed on strains cw15arg- (alias CC-4350), Elow47, UVM4, and UVM11 according to published protocols^50^. The resulting reads were mapped with bwa mem (v0.7.17-r1188) using default parameters to the sequences of the following *C. reinhardtii* transposons from NCBI: Bill (DQ446204.1), Gulliver (AF019750.1 and AF019751.1), MRC1 (DQ446210.1), Pioneer1 (U19367.1), REM1 (AY227352.1), Tcr1 (DQ446205.1), Tcr3 (Y14652.1 and Y14653.1), TOC1 (X56231.1), and TOC2 (X84663.1). The resulting alignment files were filtered to isolate half-mapped reads (i.e., those with one end mapped and one end unmapped) with samtools (v 1.9-58-gbd1a409). The remaining subset of half-mapped reads was then converted back to fastq format with samtools, and mapped to the *C. reinhardtii* genome assembly (v5; phytozome.net) with bwa mem, as before. The resulting alignment files were merged with samtools, and used to produce a bed file of total coverage with bedtools (v2.29.2) genomecov. Coverage data were used to identify the coordinates of transposons in these four strains as regions with at least 10 half-mapped reads. Next, the number of half-mapped reads from each strain was quantified with bedtools to determine the coverage for each transposon. These counts were combined into a single data table for further analysis in R. Each transposon at each locus in each strain was scored as present if ≥10 reads mapped to it. To identify loci with transposons that differ between the four strains, the ratio of counts for the strain with the most counts versus the fewest counts was calculated for each locus. Those loci with a ratio ≥10 were validated by manual curation in IGV version 2.8.10 (https://igv.org/).

### RNAseq and data analysis

For RNAseq analysis, algal strains were grown in liquid TAP medium with the revised nutrient supplementation^45^ under continuous light (90 µE m^−2^ s^−1^). Once cells reached a concentration of 5–6 × 10^6^ cells mL^−1^, a total of 5 × 10^7^ cells were collected by centrifugation at 1424 × *g* for 4 min at 4 °C. Total RNA was extracted with the TRIzol reagent as previously described^20^ with the following modifications. RNA was treated with DNase (Zymo Research), followed by a cleaning and concentration step with the RNA Clean & Concentrator-5 kit (Zymo Research). RNA quality control and library preparation were performed by the Functional Genomics Laboratory (FGL), California Institute for Quantitative Biosciences (QB3), Berkeley. Samples were evaluated for quality on a 2100 expert Plant RNA Pico microfluidic chip on a Bioanalyzer 2100 (Agilent). Enrichment of poly(A) mRNA and library preparation was performed using the KAPA mRNA HyperPrep kit (Roche). Sequencing of the samples was performed on an Illumina HiSeq4000 by the Vincent J. Coates Genomics Sequencing Laboratory at UC Berkeley.

The sequenced reads were aligned to the *C. reinhardtii* reference genome (v.5) available from Phytozome (http://phytozome.net) by means of RNA-STAR (v2.4.0j). Default settings were used except for --alignIntronMax 3000. Aligned reads were then assigned to *C. reinhardtii* gene models (v.5.5) with the featureCounts program of the Rsubread package (v1.12.6) within the R statistical computing platform (v4.0.1). The resulting counts per gene were then used to identify DEGs and to plot a principal component analysis with the DESeq2 package (v1.18.1; https://bioconductor.org/packages/release/bioc/html/DESeq2.html) in R. Principal component analysis was performed in R with the prcomp function on the regularized log-transformed counts for the 1000 genes with the greatest variance, and plotted with ggplot2 (v3.3.2). MA plots were generated with the plotMA function in DESeq2 with alpha = 0.01 and lfcThreshold = 1. Log_2_ fold-change shrinkage was calculated with the apeglm method^53^ in R. A Venn diagram of DEGs was plotted with the VennDiagram package (v1.6.20) in R. Aligned reads were used to calculate normalized transcript abundances in terms of fragments per kbp of gene per million reads (FPKMs) with cuffdiff (v2.2.1). The density of FPKMs for the transcriptome of each strain was plotted with cummeRbund (v2.24.0) in R.

The RNAseq data, including raw reads and FPKM expression tables, were deposited in the NCBI Gene Expression Omnibus (GEO) database under accession GSE128981.

### Protein homology and phylogenetic analyses

Gene and protein BLAST analyses were performed using the Phytozome web page (Phytozome v10, v11 or v12.1; http://www.phytozome.net/) and the NCBI database. The *Saccharomyces* genome database (http://www.yeastgenome.org/) and the HGNC (http://www.genenames.org/cgi-bin/genefamilies/set/937) database were used to extract all annotated histone deacetylases from yeast and humans, respectively. Sequences annotated as histone deacetylases in *Arabidopsis* and *Chlamydomonas* were identified in the Phytozome database v11 and v12.1. The *Arabidopsis* set was identical to that reported in a previous study^24^. Amino acid sequences of sirtuin family members from other organisms were obtained from NCBI (https://www.ncbi.nlm.nih.gov/).

For phylogenetic analysis of HDAC sequences, multiple sequence alignments were performed with ClustalW^54^ and neighbor-joining phylogenetic analysis was conducted with the MEGAX software (Molecular Evolutionary Genetics Analysis, https://www.megasoftware.net/mega4/). For phylogenetic tree construction, sirtuin sequences from model organisms representing different taxonomic groups were used: *Chlamydomonas reinhardtii* (CrSRTA-C; Cre10.g462200.t1.2, Cre12.g524650.t1.2, Cre05.g234663.t1.1), *Saccharomyces cerevisiae* (ScSir2p, ScHst1-4; YDL042C, YOL068C, YPL015C, YOR025W, YDR191W), *Drosophila melanogaster* (DmSirt1, 2, 4, 6, and 7; NP_477351.1, NP_001287422.1, NP_572241.2, NP_649990.2, NP_651664.2), *Danio rerio* (DrSIRT1-7; XP_005173091.1, NP_955890.1, NP_001073643.1, XP_005166125.1, XP_005170200.1, XP_021335349.1, XP_001336438.3), *Homo sapiens* (HsSIRT1-7; NP_036370.2, NP_036369.2, NP_036371.1, NP_036372.1, NP_036373.1, NP_057623.2, NP_057622.1), *Arabidopsis thaliana* (AtSRT1 and AtSRT2; AT5G55760.1, AT5G09230.7), the protozoan *Trypanosoma brucei* (TbSIR2rp1-3; Tb427_070021200.1, Tb427_080035900.1, Tb427_040027700.1), and the bacteria *Escherichia coli* (EcCobB; ANK06644.1) and *Streptococcus pneumoniae* (SpCobB; CWJ68461.1). The sequences were subjected to ClustalW multiple sequence alignment with the MEGAX software. An unrooted phylogenetic tree was produced by applying the neighbor-joining method with 500 bootstrap replicates.

### Structural alignment of the Sir2 domain of SRTA isoforms

The 3D structures of the Sir2 domains of *Chlamydomonas* SRTA and the mutated versions of SRTA from UVM11 (SRTA_UVM11_, P222L) and UVM4 (SRTA_UVM4,_ G55S) were predicted by homology modeling, based on the published 3D structure of human SIR6 (5mf6A) using ModBase (http://modbase.compbio.ucsf.edu/modbase-cgi/index.cgi). The modeled residues comprise positions 17 to 272 of the wild-type SRTA protein and amino acids 16 to 276 of the template protein SIRT6 (46% identity). The output structures were visualized by Chimera 1.12 (http://www.cgl.ucsf.edu/chimera).

### Validation of *srta-3* and *smm12* insertion mutants

Insertion mutants in loci *srta* (Cre10.g462200, mutant allele *srta-3*) and *smm12* (Cre03.g200350) were ordered from the CLiP mutant library^32^ and the insertions were verified by PCR and Southern blot analysis. The primers used are listed in Supplementary Table 10. For Southern blotting, DNA samples from single cell-derived *srta-3* mutant lines were digested with AhdI and PsiI, and hybridized to a *SRTA*-specific probe. A probe hybridizing to the 5′ end of the *SRTA* gene was generated by digesting plasmid pJR81 with ClaI and NheI, and purifying the DNA fragment of interest after gel electrophoretic separation. In addition, DNA of *srta-3* mutants was digested with XhoI and HindIII, and hybridized to a probe specific for the 5′ end of the *SMM12* gene. This probe was generated by PCR using primers oJN29 (5′-TTGCGAACACTTCGGGCGAT-3′) and oJN30 (5′-ACCACCCTGGACAGAACCGA-3′). Insertion of the 5′ junction of the CIB cassette into the first intron was confirmed by sequencing of the PCR amplicons. We did not succeed with mapping the exact 3′ junction of the insert, which was predicted to be located within gene Cre03.g200350 (*SMM12*) on chromosome 3. Southern blot analysis revealed an additional insertion in locus Cre03.g200350. The two insertions in Cre10g.462200 and Cre03.g200350 cannot be separated, as evidenced by analysis of T1 segregants of crosses of *srta-3_1* or *srta-3_2* with the wild-type CC-1690. This result suggests a complex genomic rearrangement, as it has been shown to occur frequently in insertion mutants of the CLiP library^32,55^. To determine *SRTA* transcript levels in the mutants, RT-PCR analysis was undertaken using primers SRT-E1-fw and SRT-E3-rv (Supplementary Table 10) that anneal in exon 1 and exon 3, respectively, and span the insertion site of the CIB cassette within intron 1.

As control strains, the insertion mutant LMJ.RY0402.153656 (*smm12*) was ordered from the CLiP library. The strain is predicted to carry an insertion of the CIB cassette in intron 6 of the *SMM12* (Cre03.g200350) gene. RT-PCR analyses were performed to determine *SMM12* transcript levels in the *srta-3* and *smm12* insertion mutants using primers oJN29 and oJN30 or oJN27 and oJN28 that anneal at the 5′ or 3′ end of the transcript, respectively (Supplementary Table 10).

### Treatments with histone deacetylase inhibitors

For trichostatin inhibition of histone deacetylases, 2 mL cultures grown in a 24-well plate were treated with either DMSO (as negative control) or with 200 ng mL^−1^ trichostatin (Sigma-Aldrich) for 20 h. For western blot analysis 5 µg of total soluble protein were loaded on SDS-polyacrylamide gels to determine YFP accumulation and the levels of acetylated histone H3 (H3ac).

For treatments with the inhibitor sirtinol, 2 mL aliquots of algal cultures grown to mid-exponential phase were transferred to a 24-well plate and treated with sirtinol (InSolutionTM sirtinol, Merck) at final concentrations of 5, 10, and 50 µM for 48 h. Control samples were treated with DMSO only. For immunoblot analysis, 5 µg of total soluble protein were loaded.

For treatments with the inhibitor OSS_128167, 1 mL cultures of *Chlamydomonas* cells were grown to mid-exponential phase in a multi-well plate and incubated with the sirtuin inhibitor OSS_128167 (Selleckchem) at a final concentration of 100 µM for 48 h. As a negative control, samples were incubated with DMSO only (i.e., 4 µl of a 25 mM OSS_128167 stock solution dissolved in DMSO, or 4 µl of DMSO were added to 1 mL cell culture). For immunoblot analysis, samples of 15 µg of extracted total soluble protein were used.

### Reporting summary

Further information on research design is available in the Nature Research Reporting Summary linked to this article.

## Supplementary information

Supplementary Information

Peer Review File

Description of Additional Supplementary Files

Supplementary Data 1

Supplementary Data 2

Supplementary Data 3

Supplementary Data 4

Supplementary Data 5

Supplementary Data 6

Supplementary Data 7

Supplementary Data 8

Reporting Summary

## Data Availability

The data supporting the findings of this study are available within the paper and its supplementary information files. A reporting summary for this article is available as a supplementary information file. The datasets and materials generated and analyzed during the current study are available from the corresponding author upon request. High-throughput sequencing data have been deposited in the National Center for Biotechnology Information Sequence Read Archive under accession numbers SRR1797981, SRR6872092, SRR6872091, and SRR6872090 for genome resequencing of strains cw15arg-, Elow47, UVM4, and UVM11, respectively. The RNAseq data, including raw reads and FPKM expression tables, were deposited in the NCBI Gene Expression Omnibus (GEO) database under accession GSE128981. Source data are provided with this paper.

## Source data

Source Data
