# Publisher Correction: An epigenetic gene silencing pathway selectively acting on transgenic DNA in the green alga *Chlamydomonasis*

**DOI:** 10.1038/s41467-020-20623-0

**Published:** 2021-01-04

**Authors:** Juliane Neupert, Sean D. Gallaher, Yinghong Lu, Daniela Strenkert, Na’ama Segal, Rouhollah Barahimipour, Sorel T. Fitz-Gibbon, Michael Schroda, Sabeeha S. Merchant, Ralph Bock

**Affiliations:** 1 Max-Planck-Institut für Molekulare Pflanzenphysiologie, Am Mühlenberg 1, D-14476 Potsdam-Golm, Germany; 2 University of California Los Angeles, Department of Chemistry and Biochemistry, and Institute for Genomics and Proteomics, 607 Charles E. Young Dr. East, Los Angeles, CA 90095 USA; 3 University of California Los Angeles, Department of Molecular, Cell and Developmental Biology, 610 Charles E. Young Dr. South, Los Angeles, CA 90095 USA; 4 Present Address: Nanjing University of Science and Technology, Institute of Chemical Biology and Advanced Materials, Xiaolingwei Street 200, 210094 Nanjing, China; 5 Present Address: University of California Berkeley, Departments of Molecular and Cell Biology and Plant and Microbial Biology, Berkeley, CA 94720 USA; 6 Present Address: Technical University of Kaiserslautern, Molecular Biotechnology & Systems Biology, Paul-Ehrlich-Straße 23, 67663 Kaiserslautern, Germany

**Keywords:** Histone analysis, Gene expression analysis, Plant biotechnology, Epigenetics

Correction to: *Nature Communications* 10.1038/s41467-020-19983-4, published online 8 December 2020.

In the original version of this Article, the *y*-axis label of Fig. 1d was incorrectly given as “Relative H3Ac/H3 levels” and should read “Relative H4Ac/H3 levels”. In addition, the probe target in the left panel of Figure 1f was misspelled as “*Guliver*” and should read “*Gulliver*”. Furthermore, the last label of the diagram on the top of Fig. 2c was given incorrectly as “PF26” and should read “PF25”. Moreover, the label of the branch pointed by the arrow symbol in cluster II of the phylogenetic tree in Fig. 3c was incorrectly labelled as “CrSRTb” and the correct label is “CrSRTB”. These errors have been corrected in both the PDF and HTML versions of the Article.

