# Supplementary material for: An epigenetic gene silencing pathway selectively acting on transgenic DNA in the green alga Chlamydomonas
Source: Nat Commun. 2020 Dec 8;11:6269. doi: 10.1038/s41467-020-19983-4 (PMC7722844; doi:10.1038/s41467-020-19983-4)
Supplement: Supplementary file 1 — Supplementary Information [file 41467_2020_19983_MOESM1_ESM.pdf]

An epigenetic gene silencing pathway selectively acting on transgenic DNA in the green alga *Chlamydomonas*

Neupert *et al.*

Supplementary Table 1. High-resolution mapping of *uvm* using PCR markers on chromosome 10 of the *Chlamydomonas* genome[a].

		**Markers on Linkage Group X**							
**Segregant**	**YET [%]**	CNA83	ACE6301	FLU	X1	X2	X3	X4	PF25
I 4c	53								
I 8a	63								
I 11a	35								
I 18a	45								
I 23a	40								
II 51b	35								
II 63a	62								
II 70b	63								
II 71a	68								
II 75b	41								
II 77b	45								
II 79a	37								
I 21a	74								
I 44a	40								
I 47a	83								
I 59c	85								
I 60a	35								
I 61c	41								
I 7a	50								
I 20c	37								
I 21b	47								
I 22a	42								
I 26c	32								
I 30a	60								
II 44a	33								
I 66a	35								
I 81a	56								
I 83a	85								
I 84a	70								
I 107a	55								
I 111a	100								

[a] Amplicons corresponding to the allele of strain UVM11-CW are indicated by blue boxes, amplicons representing the CC-2290 allele are in orange. White boxes indicate that no PCR analysis was performed or no amplicon was obtained. YET: YFP-expressing transformants.

Supplementary Table 2. Summary of the fine mapping analysis of the *uvm* locus[a].

PCR marker	CNA83	ACE6301	FLU	X1	X2	X3	X4	PF25
Number of segregants analyzed	17	18	31	31	25	31	31	30
Segregants with UVM11 marker	15	16	27	31	25	30	30	26
Linkage to *uvm* [cM]	11.8	11.1	12.9	0	0	3.2	3.2	13.3
Distance to *uvm* [kb]	1176	1111	1290	0	0	323	323	1333

[a] PCR markers localized on chromosome 10 and their linkage to *uvm* are given. Physical distances were calculated based on the assumption that 1 cM is, on average, equivalent to 100 kb in the *Chlamydomonas* nuclear genome[36].

Supplementary Table 3. Summary of the genetic variants detected by whole-genome sequencing of the expression strains, UVM4 and UVM11, relative to the wild type-like parental strain Elow47[a].

	**UVM4**	**UVM11**
**SNVs**	160	214
**InDels**	61	46
**All**	221	260

[a] The numbers of variant loci, including single nucleotide variants (SNVs) and insertions/deletions (InDels), that are specific to UVM4 or UVM11 are given.

Supplementary Table 4. Overview of the predicted effects of the genetic variants identified in strains UVM4 and UVM11.

	UVM4	UVM11
**Altered stop codon**	1	1
**Frame-shift InDel**	3	2
**In-frame InDel**	10	3
**Missense codon**	23	30
**Synonymous codon**	16	24
**Non-coding**	168	200
**Total**	221	260

Supplementary Table 5. Genes harboring distinct non-synonymous mutations in both UVM4 and UVM11, and their functional annotations (http://phytozome.jgi.doe.gov/).

**Gene ID**	**Gene name**	**Functional annotation**
Cre10.g462200	*HDA17* (renamed to *SRTA*)	K11416 - mono-ADP-ribosyltransferase sirtuin 6 (SIRT6, SIR2L6)
Cre15.g638150	*OPR72*	PF08373 - RAP domain (RAP)

Supplementary Table 6. List of *Chlamydomonas reinhardtii* genes annotated as histone deacetylases[a].

Gene	New name	Closest homolog in *A.t.*	*A.t.* locus	Similarity [%]
Cre03.g158200	HDA1	HDA2	AT5G26040	41.1
Cre03.g160000	HDA2	HDA15	AT3G18520	9.6
Cre03.g162050	HDA3	HDA15	AT3G18520	23
Cre05.g234663	SRTC	SRT1	AT5G55760	7.5
Cre06.g277350	HDA4	HDA19 (AtRPD3A)	AT4G38130	58.5
Cre06.g290400	HDA5	HDA14	AT4G33470	40.4
Cre08.g358555	HDA6	HDA2	AT5G26040	21.1
Cre09.g387393	HDA7	HDA19 (AtRPD3A)	AT4G38130	53
Cre10.g462200	SRTA	SRT1	AT5G55760	33.5
Cre11.g467706	HDA8	HDA5	AT5G61060	26.7
Cre11.g467751	HDA9	HDA19 (AtRPD3A)	AT4G38130	21
Cre11.g467785	HDA10	HDA5	AT5G61060	33.4
Cre11.g469600	HDA11	HDA5	AT5G61060	23.8
Cre11.g477800	HDA12	HDA14	AT4G33470	14.2
Cre12.g524650	SRTB	SRT2	AT5G09230	34.9
Cre14.g632300	HDA13	HDA2	AT5G26040	27.6
Cre16.g673150	HDA14	HDA9	AT3G44680	75.4

[a] Genes were extracted from the Phytozome v12.1 database (by keyword searches with "histone deacetylase" and "sirtuin"). From the 17 genes found, three are predicted to be of the Sir2 type: SRTA (Cre10.g462200), SRTB (Cre12.g524650) and SRTC (Cre05.g234663). The other 14 genes show homology to the RPD3/HDA1-type of HDACs and were named accordingly. The closest homolog found in *Arabidopsis* (*A.t.*), including the gene name and the similarity at the protein level, is also given.

Supplementary Table 7. Efficiency of YFP expression in *srta-2* transformants of expression strains UVM4 and UVM11[a].

pJR85 transformant	1	2	3	4	5	6	7	8	9	10	11	12
YET [%] pJR85; UVM11	55	55	45	40	70	80	65	71	85	55	45	45
YET [%] pJR85; UVM4	60	70	65	55	65	55	50	35	50	55	55	55

[a] Expression strains UVM4 and UVM11 were transformed with plasmid pJR85 (Fig. 4a) encoding the *srta-2* mutant allele. Each individual *srta-2* transformant was supertransformed with the *YFP* reporter gene to determine its YFP expression capacity. The YFP expression capacity is given as YET in percent and gives the number of *YFP* supertransformants that showed strong YFP fluorescence relative to the number of total *YFP* supertransformants analyzed for each *srta-2* line.

Supplementary Table 8. Assessment of the transgene expression capacity in the *srta-3* mutant strains by supertransformation with a *YFP* transgene[a].

Strain	Genotype	YET [%]
srta-3_1	*srta / smm12*	8.3
srta-3_3	*srta / smm12*	36.1
CC1690 x srta-3_1_T1-1B	*srta / smm12*	19.4
CC1690 x srta-3_1_T1-2A	*srta / smm12*	19.1
CC1690 x srta-3_3_T1-3B	*srta / smm12*	47.2
CC1690 x srta-3_3_T1-4B	*srta / smm12*	30.6
CC5325 x CC1690_T1-1	*SRTA/ SMM12*	2.8
CC5325 x CC1690_T1-2	*SRTA/ SMM12*	0
CC1690 x srta-3_3_T1-1A	*SRTA/ SMM12*	2.8
UVM11	*uvm11/ SMM12*	40.0
Elow47	*SRTA/SMM12*	0

[a] Expression strengths of *YFP* are given as YET in percent (number of strongly expressing YFP lines relative to the total number of transformants analyzed). YFP expression was determined in the *srta-3* strains 1 and 3, and the wild type CC-5325 as control. In addition, individual segregants from the T1 generation of crosses of *srta-3*_1 or *srta*-3_3 with the wild type CC-1690 were included. The genotype of the strains analyzed is given, with *SRTA* representing the wild-type allele and *srta-3* being the mutant allele carrying the insertion. The mutated allele in mutant strain UVM11 is named *uvm11* (*srta-1*). Source data are provided as a Source Data file.

Supplementary Table 9. Overview of transformation experiments conducted in the course of this study[a].

**Strain**	**Genotype**	**Vector**	**Gene**	**RE**	**Selection**
CC-4350	*SRTA*	pRMB12	*CrYFP*	AhdI	Paro
Elow47	*SRTA*	pRMB12	*CrYFP*	AhdI	Paro
UVM4	*srta-2*	pRMB12	*CrYFP*	AhdI	Paro
UVM11	*srta-1*	pRMB12	*CrYFP*	AhdI	Paro
Elow47	*SRTA*	pJR39	*vYFP*	KpnI	Paro
UVM4	*srta-2*	pJR39	*vYFP*	KpnI	Paro
UVM11	*srta-1*	pJR39	*vYFP*	KpnI	Paro
UVM11-CW x CC-2290 progeny	*srta-1*	pJR39	*vYFP*	KpnI	Paro
UVM4	*srta-2*	pJR81	*UVM11*	PvuI	Hyg *
UVM11	*srta-1*	pJR81	*UVM11*	PvuI	Hyg *
UVM4	*srta-2*	pJR88	*UVM11-FLAG*	PvuI	Hyg *
UVM11	*srta-1*	pJR88	*UVM11-FLAG*	PvuI	Hyg *
UVM4	*srta-2*	pJR85	*uvm4*	PvuI	Hyg *
UVM11	*srta-1*	pJR85	*uvm4*	PvuI	Hyg *
UVM4-pJR81	*srta-2/SRTA*	pJR39	*YFP*	KpnI	Paro
UVM11-pJR81	*srta-1/SRTA*	pJR39	*YFP*	KpnI	Paro
UVM4-pJR88	*srta-2/SRTA*	pJR39	*YFP*	KpnI	Paro
UVM11-pJR88	*srta-1/SRTA*	pJR39	*YFP*	KpnI	Paro
UVM4-pJR85	*srta-2/srta-2*	pJR39	*YFP*	KpnI	Paro

UVM11-pJR85	*srta-1/srta-2*	pJR39	*YFP*	KpnI	Paro
UVM4-pJR39	*srta-2/YFP*	pJR81	*UVM11*	PvuI	Paro
UVM11-pJR39	*srta-1/YFP*	pJR81	*UVM11*	PvuI	Hyg *
srta-3	*srta-3*	pJR91	*YFP*	AhdI	Hyg
smm12	*SRTA*	pJR91	*YFP*	AhdI	Hyg

[a] Transformations marked with an asterisk indicate transformants that were maintained under selective conditions on solid medium to minimize the risk of loss of transgene expression. RE: restriction enzyme used for vector linearization, Gene: transgene introduced., Paro: selection on 10 µg mL^{-1} paromomycin, Hyg: selection on 10 µg mL^{-1} hygromycin.

Supplementary Table 10. Primer sequences used in this study.

Experiment	Target	Primer 1	Sequence 5'-3'	Primer 2	Sequence 5'-3'
ChIP	*CRY1-1* transcription start site	CRYRBCSFor1	CAGAGGCACGAGAGCTCCCGT	UTRCRYrev1	CATCGACCAGGTGCGAACGC
	YFP transcription start site	Ppsadfor3	CTTGTGGCCGTTTACGTCG	Ppsadrev3	AGCAAGCCAGGGTTAGGTGTT
	endogenous *CYC6*	PCYC6for	ACACGCCCCTCATTCACAGA	PCYC6rev	GCACACGAGACACTCCGAGC
	telomere-flanking region	TFR1for	GGGTTTTGCAGGGTTTGGA	TFR1rev	CCTCATCATGGTCACCCACA
	endogenous *PSAD*	PSADpromFor1	CCAGGGTTAGGTGTTGCGCTC	PSADpromRev1	GATGAAGCGCGAGTGGCAG
Test for integration of *YFP* 5' end	*YFP*	M13f	GTAAAACGACGGCCAGT	5'PSADrev	CGAGCCCTTCGAACAGCCAGG CCG
Test for presence of *SRTA* (of pJR81/pJR88)	*SRTA* transgenes	M13f	GTAAAACGACGGCCAGT	X2-fw	CTGTGTTGGGATGCGGATTG
New markers for mapping	X1	X1-fw	GTCCATTCAGGCAGGGTGAT	X1-rv	CAACACAAATCGTGCCCCTG
	X2	X2-fw	CTGTGTTGGGATGCGGATTG	X2-rv	CACCTCGTCCTCCAACCATT
	X3	X3-fw	CAGGAATAGGCGTAGGCGTC	X3-rv	GGCATAACGCTTCTCACTGC
	X4	X4-fw	GGGCAACCTCAACTGACTGA	X4-rv	TCCAGGTTCACAACGTGACA
Sequencing of Cre10.g462200	Cre10.g462200	X-c3-fw	CAGCCTTCCTTTCTCTCCCG	X-c3 rv	GCACCATGTGTGTGAAGCTG
		X-c3 fw2 b	AGCCGCCTTGAACACCAAT	X-c3 rv2b	ACTACGCGACACAACACGAC
		X-c3 fw3	CCCCCAATGTGACAAACACAC	X2-rv	CACCTCGTCCTCCAACCATT
Detection of *srta-1* mutation	*SRTA* or *srta-1* allele	X-c3fw3	CCCCCAATGTGACAAACACAC	X-c3rv2b	ACTACGCGACACAACACGAC
Priming of cDNA synthesis to measure ratio of *srta : SRTA*	*SRTA* and *srta-1* cDNA	SRT3402r2	GCTCGCCGGTCCTTGG		
Detection of *srta-2* point mutation	*srta-2* cDNA of UVM4	SRT-E1-fw	CCAGGACCTCGACGACATCA	SRT-E3-rv	CGCGTACTCAAAGGGCGTG
Detection of *srta-1* point mutation	*srta-1* cDNA of UVM11	SRTE5f	AGCTGGTGGACAACATCCTG	SRTE8r	GCGTCTTCTGCAGGTTCAC
RT-PCR (wild type and *srta-3*)	*srta*	SRT-E1-fw	CCAGGACCTCGACGACATCA	SRT-E3-rv	CGCGTACTCAAAGGGCGTG
	smm12	oJN27_LC03.G7	ATCCTGTGGGGCTAGTGAGT	oJN28_LC03.G8	ACGTCACTTCCACGATCACC
RT-PCR (wild type and *smm12*)	*smm12*	oJN29_LC03.G9	TTGCGAACACTTCGGGCGAT	oJN30_LC03.G10	ACCACCCTGGACAGAACCGA
Mating type determination	*FUS1*, mt+	FUS1-up	ATGCCTATCTTTCTCATTCT	FUS1-low	GCAAAATACACGTCTGGAAG
	MID, mt-	mid-up	ATGGCCTGTTTCTTAGC	mid-low	CTACATGTGTTTCTTGACG

APHVIII of CIB cassette	CIB	oJN25-CIBfw	GCTGCGAGTGGGTTGTGGTT	oJN26-CIBrv	GTCAACCGAGCCTTCAGCGA
Test for *SRTA* allele	*SRTA*	X-c3fw	CAGCCTTCCTTTCTCTCCCG	X-c3rv	GCACCATGTGTGTGAAGCTG
5' junction of insert in *srta-3*	*SRTA*/CIB	X-c3fw	CAGCCTTCCTTTCTCTCCCG	oJN14-C1	GCACCAATCATGTCAAGCCT
Probe for *SMM12*	3'end of *SMM12*	oJN27_LC03.G7	ATCCTGTGGGGCTAGTGAGT	oJN28_LC03.G8	ACGTCACTTCCACGATCACC
	5'end of *SMM12*	oJN29_LC03.G9	TTGCGAACACTTCGGGCGAT	oJN30_LC03.G10	ACCACCCTGGACAGAACCGA

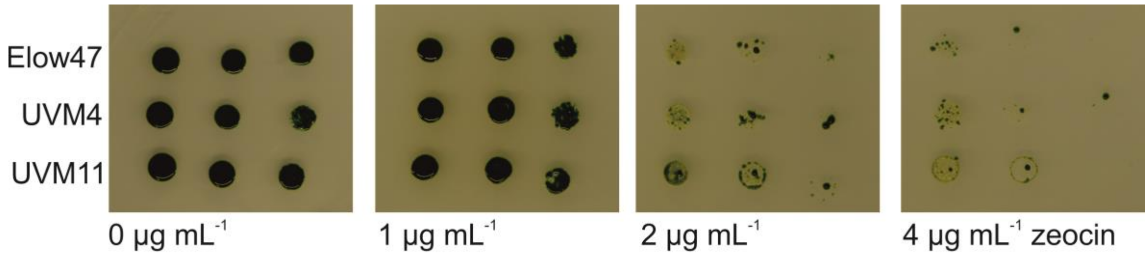

Supplementary Figure 1. Zeocin sensitivity assay of strains UVM4, UVM11 and control strain Elow47. Cells of a late-exponential culture were diluted to three different concentrations (1 x 10^7, 1 x 10^6 and 2 x 10^5 cells mL^{-1}; from left to right) and samples of 8 µL from each dilution were dropped onto TAP agar plates containing different concentrations of the DNA double-strand break-inducing agent zeocin (0, 1, 2 and 4 µg mL^{-1}). Photos of the drop tests were taken after ten days.

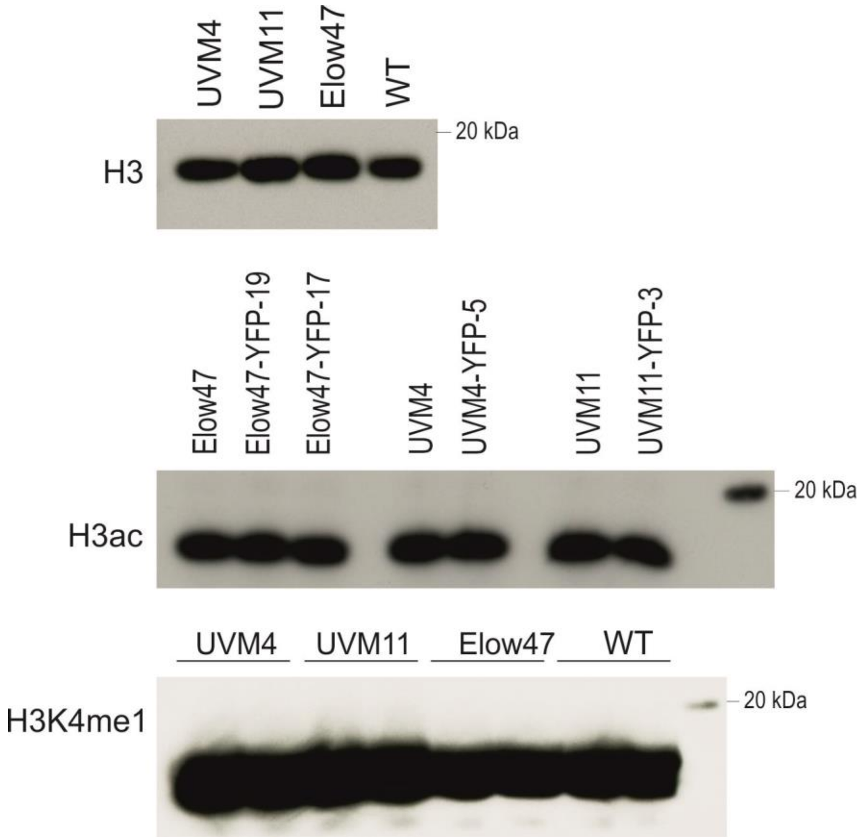

Supplementary Figure 2. UVM strains show no change in the overall level of histone 3, histone acetylation and methylation. Immunoblot analysis of total soluble protein samples was performed with antibodies against total histone 3 (H3), acetylated histone 3 (H3ac), and monomethylated histone H3 at lysine 4 (H3K4me1) in mutant and control strains transformed with the *YFP* reporter gene. Molecular weights were deduced from co-migrating protein markers and are given at the right in kDa. Each immunoblot experiment was performed at least twice with similar results obtained. Source data are provided as a Source Data file.

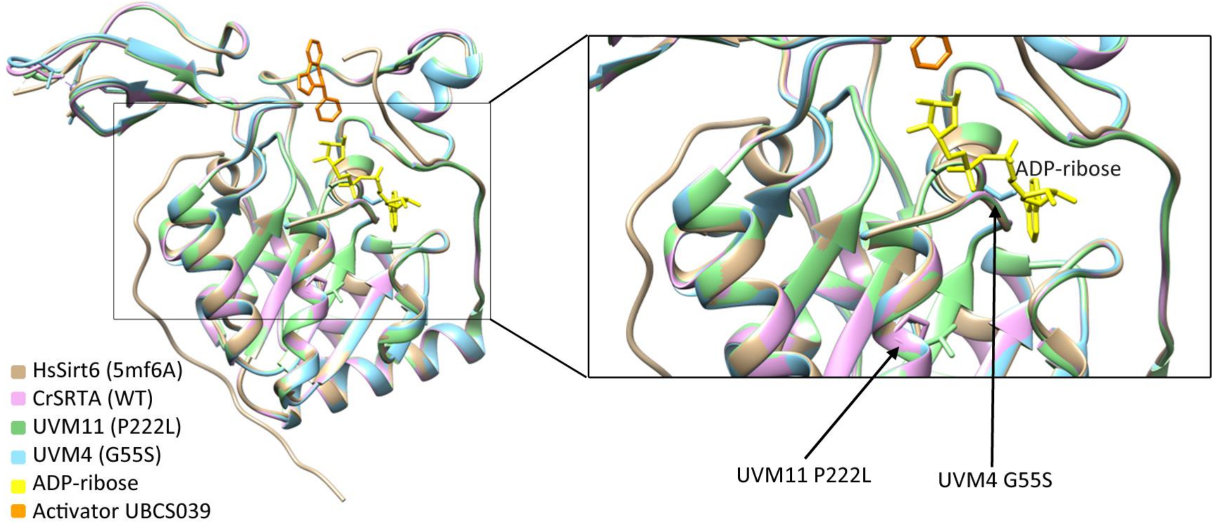

Supplementary Figure 3. Homology modeling of the *Chlamydomonas* SRTA protein (CrSRTA) from the wild type (WT) and the mutant strains UVM11 and UVM4 based on the 3D structure of the *Homo sapiens* ortholog Sirt6 (HsSirt6, 5mf6A). Modeling was performed using ModBase, using the CrSRTA amino acid sequence between positions 17 and 272, and the region between amino acids 16 and 276 of the template protein (sharing 46% identity). The output structures were visualized by Chimera 1.12. These structures showed an RMSD of 0.3. The solved structure of 5mf6A contains ADP-ribose as an NAD(+) analogue and an activator molecule (UBCS039) which together indicate the active site of the SRT domain. The amino acid substitutions in the mutated proteins from strains UVM11 and UVM4 are indicated by arrows. The G55S exchange in UVM4 affects the catalytic center. The P222L substitution in UVM11 may affect the protein at the structural level and/or disturb its interaction with other proteins.

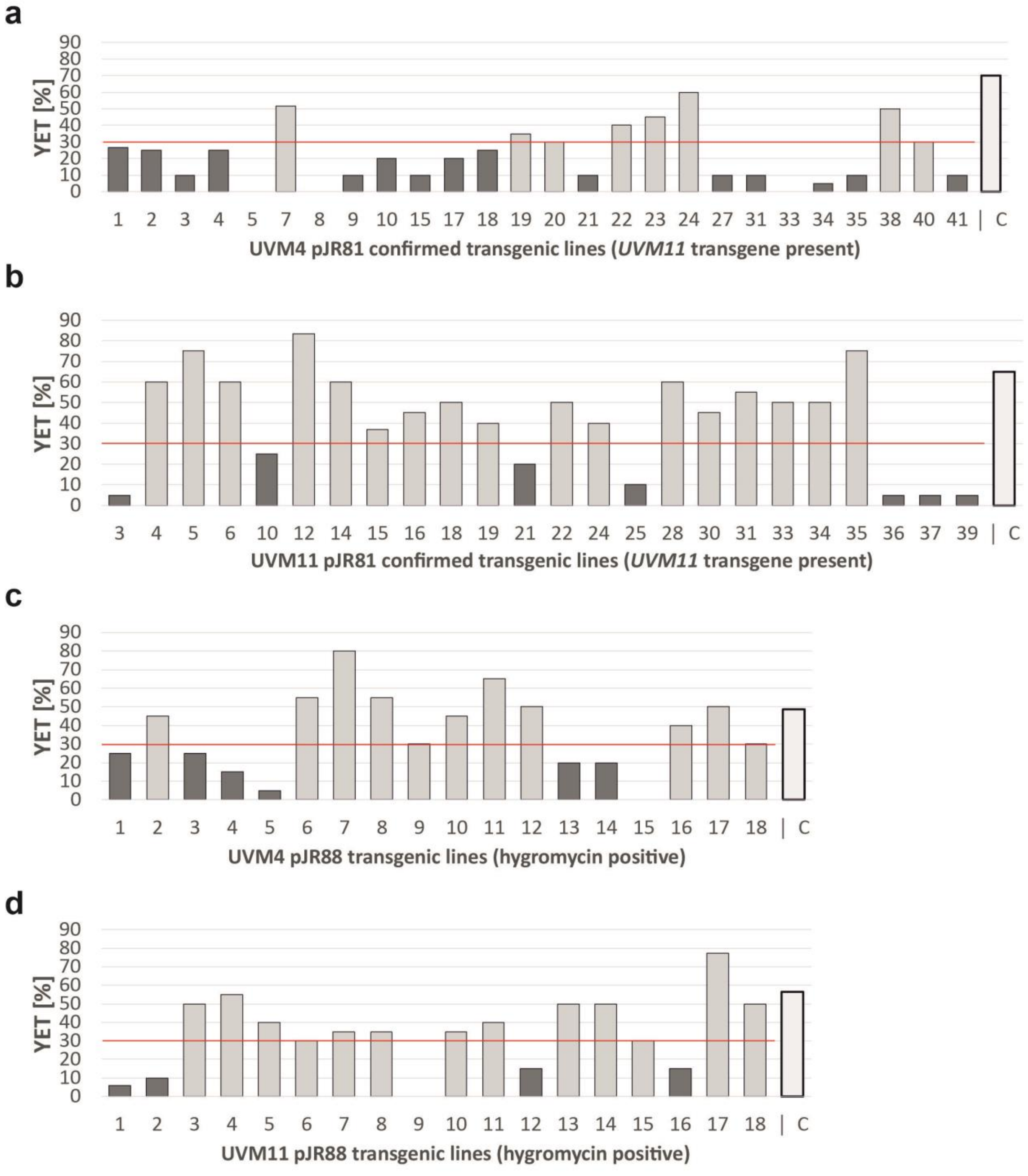

Supplementary Figure 4. Loss of transgene expression efficiency in *SRTA* transformants of *uvm* mutants. Expression strains UVM4 (**a**) and UVM11 (**b**) were transformed with plasmid pJR81 (Fig. 4a) encoding the *SRTA* wild type allele. Each pJR81 transformant carries the complete cassette of *SRTA* (as revealed by genotyping by PCR), and was supertransformed with the *YFP* reporter gene. In addition, UVM4 (**c**) and UVM11 (**d**) were transformed with

plasmid pJR88 (Fig. 4a) that contains the FLAG-tag sequence fused to the *SRTA* wild-type sequence. Individual pJR88 transformants were randomly picked after hygromycin selection and supertransformed with the *YFP* reporter gene (along with the paromomycin selectable marker) to determine their YFP expression capacity. The YFP expression capacity is given as YET (YFP-expressing transformants) in percent and gives the number of *YFP* supertransformants that showed strong YFP fluorescence relative to the number of total *YFP* supertransformants (≥20 supertransformants per line) analyzed for each *SRTA* line. *SRTA* transformants with a YET value below 30% (red line) were scored as phenotypically complemented (dark grey bars). C: control strain (not transformed with *SRTA*).

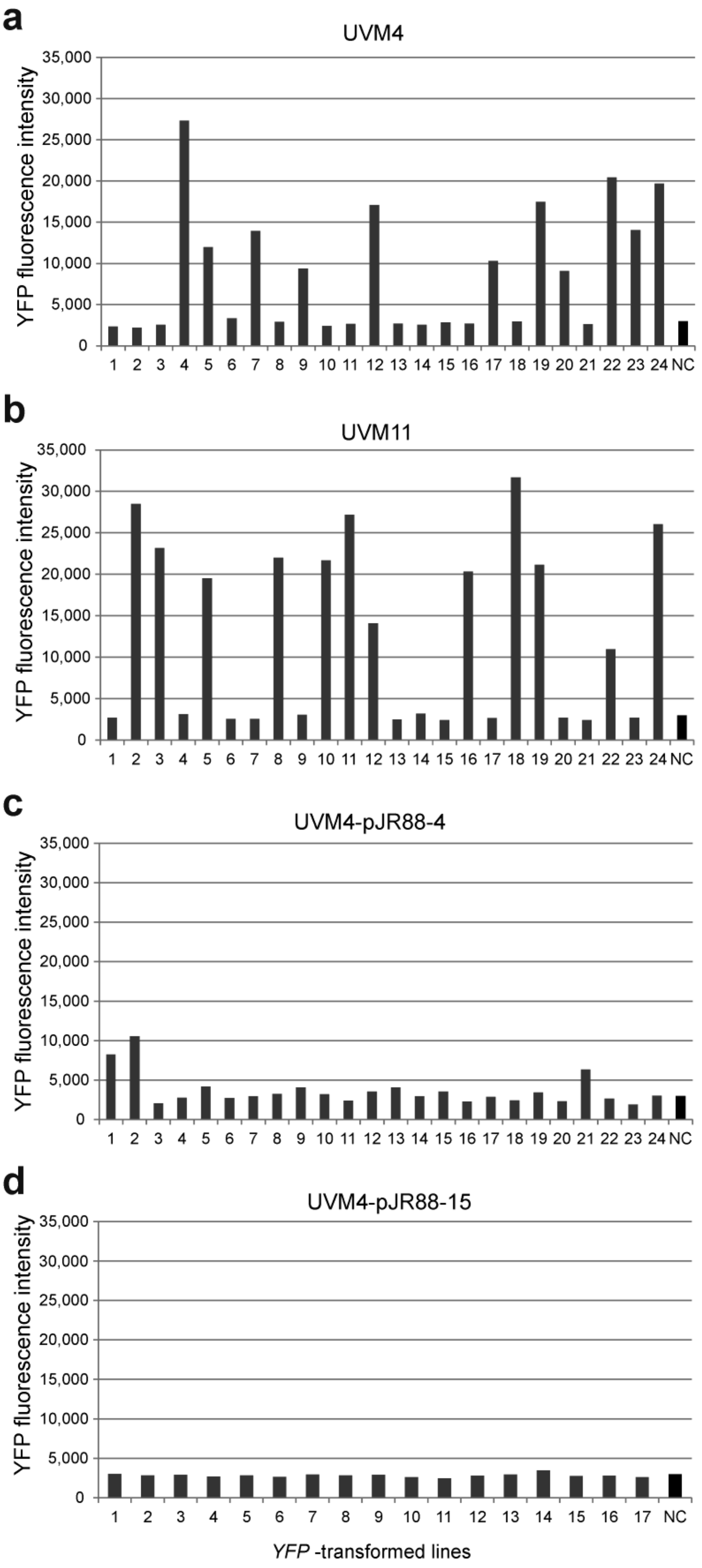
a
UVM4
b
UVM11
c
UVM4-pJR88-4
d
UVM4-pJR88-15
YFP fluorescence intensity
0
5,000
10,000
15,000
20,000
25,000
30,000
35,000
1 2 3 4 5 6 7 8 9 10 11 12 13 14 15 16 17 18 19 20 21 22 23 24 NC
1 2 3 4 5 6 7 8 9 10 11 12 13 14 15 16 17 NC
YFP -transformed lines

Supplementary Figure 5. Loss of transgene expression capacity in the UVM4 strain complemented with the *SRTA-FLAG* construct (pJR88; Fig. 4a). Two independently generated complemented strains (UVM4-pJR88-4 and UVM4-pJR88-15) are exemplarily shown. Transgene expression capacity was determined by analyzing the YFP fluorescence intensity of approximately 20 randomly selected colonies supertransformed with a *YFP* cassette using a microplate reader. The YFP fluorescence intensity was normalized to the absorbance of cultures measured at 750 nm. Cultures of non-transformed strains (UVM4 or UVM11) were included as negative controls (NC). In contrast to the expression strains UVM4 (**a**) and UVM11 (**b**) that show strong YFP fluorescence in approximately half of the putative transformants (fluorescence intensity >5000 arbitrary units), only three supertransformants of strain UVM4-pJR88-4 had YFP fluorescence intensities slightly above 5000 units (**c**) and none of the *YFP* supertransformants of the complemented strain UVM4-pJR88-15 displayed above-background YFP fluorescence (**d**).

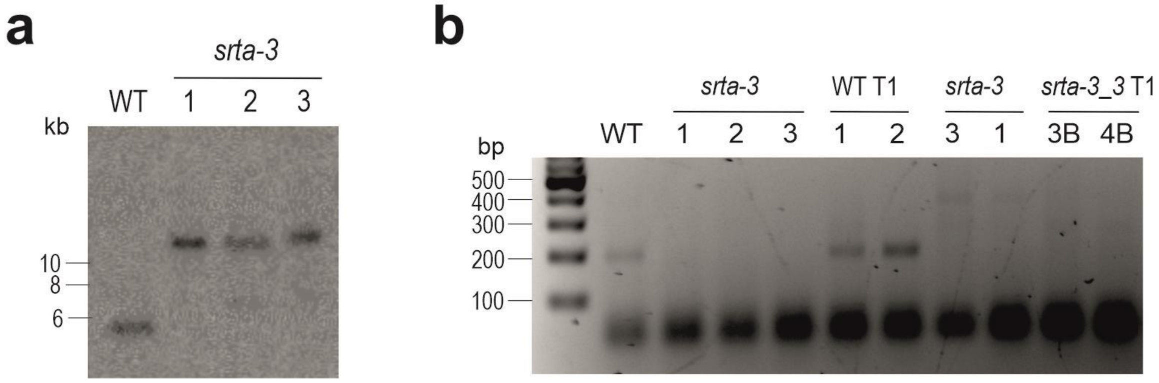

Supplementary Figure 6. The *srta-3* insertion mutant has a disrupted *SRTA* locus and shows enhanced transgene expression capacity. **a** Southern blot analysis of the *srta-3* mutant strains 1, 2 and 3 (derived from single cells of insertion mutant LMJ.RY0402.148523), and the wild-type strain CC-5325 (WT) using a *SRTA*-specific probe. Samples that appear twice in the gel represent biological replicates. 10 µg total DNA from each strain were digested with the restriction enzymes AhdI and PsiI and separated by agarose gel electrophoresis. **b** RT-PCR analysis of the *srta-3* mutant lines using primers spanning the insertion site of the CIB cassette within the first intron of the *SRTA* gene. CC-5325 (WT) and two segregants of the cross CC-5325 x CC-1690 (WT T1-1 and -2) were used as wild type-controls. In addition to the three clones of mutant strain *srta-3*, two strains (*srta-3* T1-3B and *srta-3* T1-4B) derived from the T1 progeny of the cross *srta-3*_3 x CC-1690 (and tested positive for the *srta-3* allele) were included in the analysis. The experiment was performed twice, and similar results were obtained. Fragment sizes of the DNA size marker are given at the left of each gel image. Source data are provided as a Source Data file.

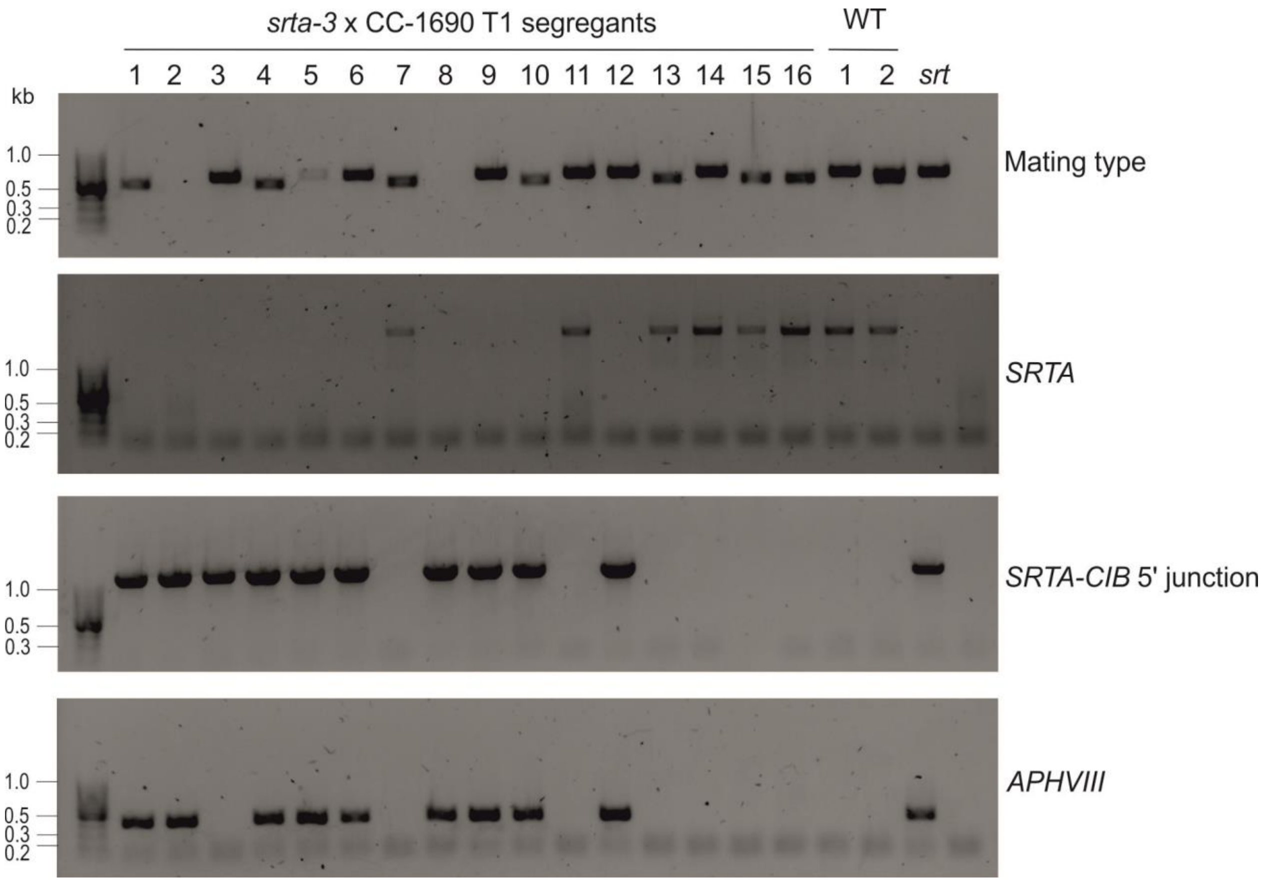

Supplementary Figure 7. PCR analysis of the T1 progeny from the cross *srta*-3_3 x CC-1690. Randomly picked segregants (numbered 1 to 16) were screened by PCR for their mating type, the presence of the *SRTA* wild type-allele, the presence of the *APHVIII* cassette and the presence of the 5' junction of the CIB cassette inserted into the intron of the *SRTA* gene. As controls, DNA of the parental *srta*-3_3 strain and two T1 segregants from the cross of wild-type strain CC-5325 with wild-type strain CC-1690 were included. Note that the mating type of the progeny segregates 1:1, as expected. For the T1 segregant line 2 of the wild-type control, PCR products of both mating types were amplified. This specific sample, therefore, is likely not a single cell-derived, haploid line, but represents either a vegetative diploid strain or a mixed culture of two non-separated segregants. Fragment sizes of the DNA size marker are given at the left in kb. Results were obtained from a singular crossing experiment with 16 independent segregants analyzed. Source data are provided as a Source Data file.

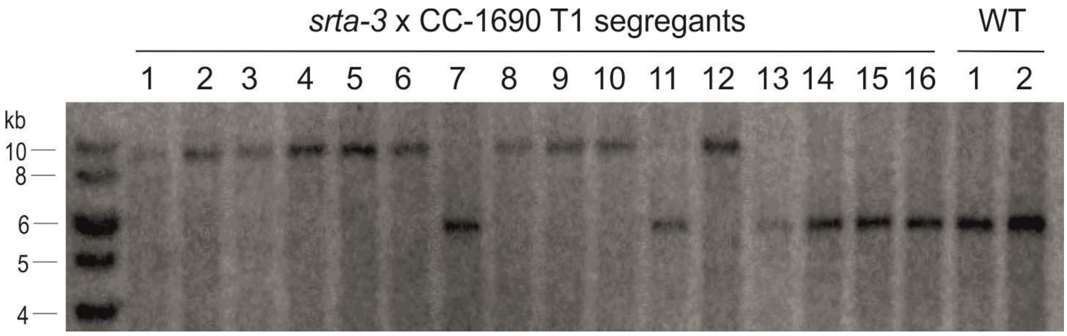

Supplementary Figure 8. Southern blot analysis of the T1 progeny from the cross *srta*-3_3 x CC-1690. DNA of randomly picked segregants (numbered 1 to 16) that had been genotyped by PCR (Supplementary Figure 7) was digested with XhoI and HindIII, and hybridized to a probe specific to the 5’ end of the *SMM12* gene. The band sizes of the molecular weight marker are given at the left in kb. Note that all segregants with the *smm12* insertion allele also carry the *srta-3* insertion allele (cf. Supplementary Figure 7). Conversely, the segregants yielding a PCR product for the *SRTA* wild-type allele (Supplementary Figure 7) do contain the *SMM12* wild-type allele. This excludes the presence of two independent insertions of the CIB cassette in the genome of the *srta-3* mutant, but rather suggests a genomic rearrangement. Fragment sizes of the DNA size marker are given at the left in kb. Source data are provided as a Source Data file.

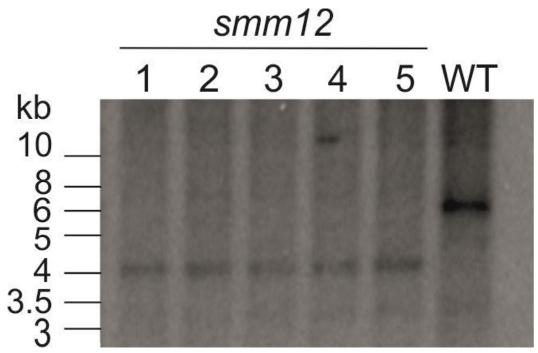

Supplementary Figure 9. Southern blot analysis of single cell-derived lines of insertion mutant *smm12*. The mutant strain carries the insertion cassette in intron 6 of the *SMM12* gene. DNA of five single cell-derived strains was digested with XmnI and HindIII, and hybridized to a probe specific to the 3’ end of the *SMM12* gene. The band sizes of the molecular weight marker are given at the left in kb. Results were obtained from a single Southern blot experiment. Source data are provided as a Source Data file.

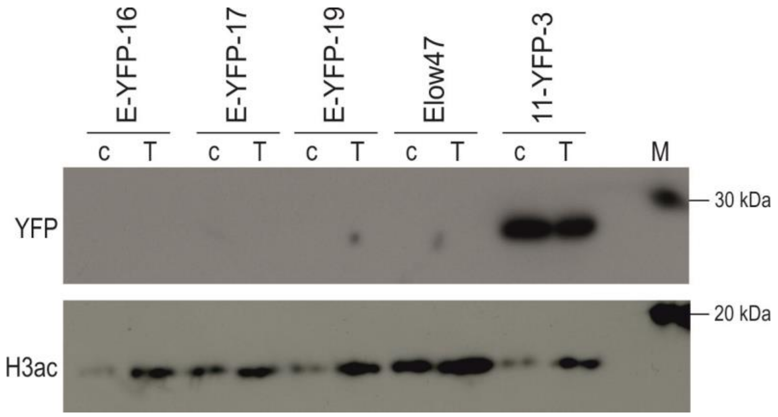

Supplementary Figure 10. Treatment of *YFP* transformants of the wild type-like strain Elow47 and the expression strain UVM11 with the histone deacetylase inhibitor trichostatin. 2 mL algal cultures grown in a 24-well plate were treated with either DMSO (as negative control; c) or 200 ng mL^{-1} trichostatin (T) for 20 hours. Immunoblot analysis of extracted total soluble protein was performed with antibodies against GFP to detect YFP accumulation, and antibodies against acetylated histone H3 (H3ac). E-YFP-16, E-YFP-17, E-YFP-19: three independent transformants of strain Elow47; Elow47: untransformed control; 11-YFP-3: transformant of expression strain UVM11. Molecular weights were deduced from co-migrating protein markers and are given at the right. Source data are provided as a Source Data file.

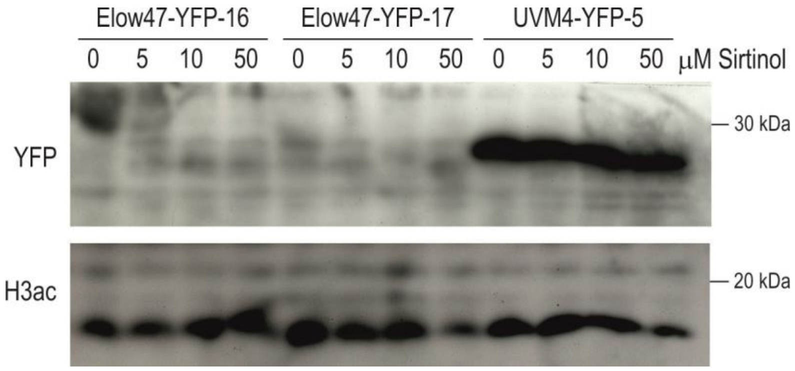

Supplementary Figure 11. Treatment of two independent *YFP* transformants of the wild type-like strain Elow47 (Elow47-YFP-16 and Elow47-YFP-17) and a *YFP* transformant of expression strain UVM4 (UVM4-YFP-5) with the sirtuin inhibitor sirtinol. After treatment with different concentrations of sirtinol (0, 5, 10, and 50 µM) for 48 h, total protein was isolated and western blot analysis was performed with antibodies against GFP to detect YFP accumulation and antibodies against acetylated histone H3 (H3ac). Molecular weights were deduced from co-migrating protein markers and are given at the right. Source data are provided as a Source Data file.

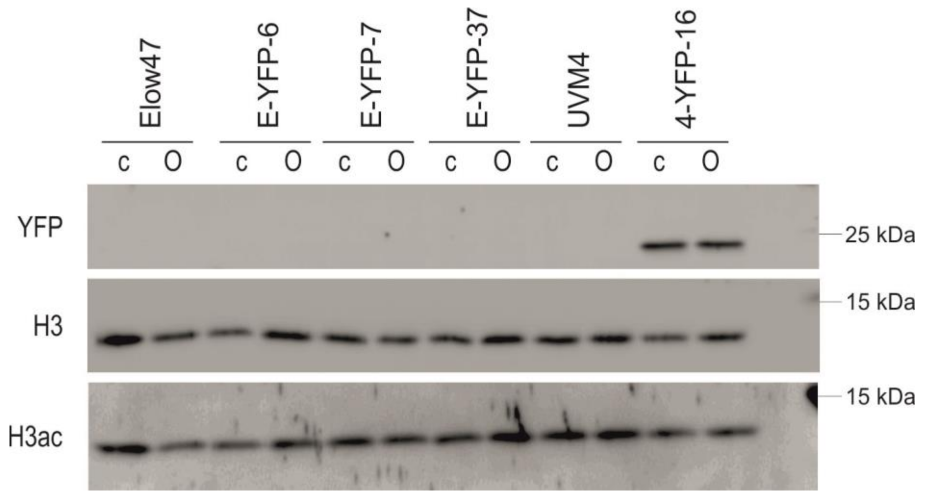

Supplementary Figure 12. Treatment of *YFP* transformants of wild type-like strain Elow47 and expression strain UVM4 with the sirtuin inhibitor OSS_128167. Cultures grown to mid-exponential phase in a 24-well plate were treated with either DMSO (as negative control; c) or 100 µM OSS_128167 (O) for 48 hours. Immunoblot analysis of total soluble protein samples was performed with antibodies against GFP (to detect YFP accumulation), histone H3 (H3), and acetylated histone H3 (H3ac). E-YFP-6, E-YFP-7, E-YFP-37: three independent transformants of strain Elow47; Elow47, UVM4: untransformed control strains; 4-YFP-16: transformant of expression strain UVM4. Molecular weights were deduced from co-migrating protein markers and are given at the right. Source data are provided as a Source Data file.

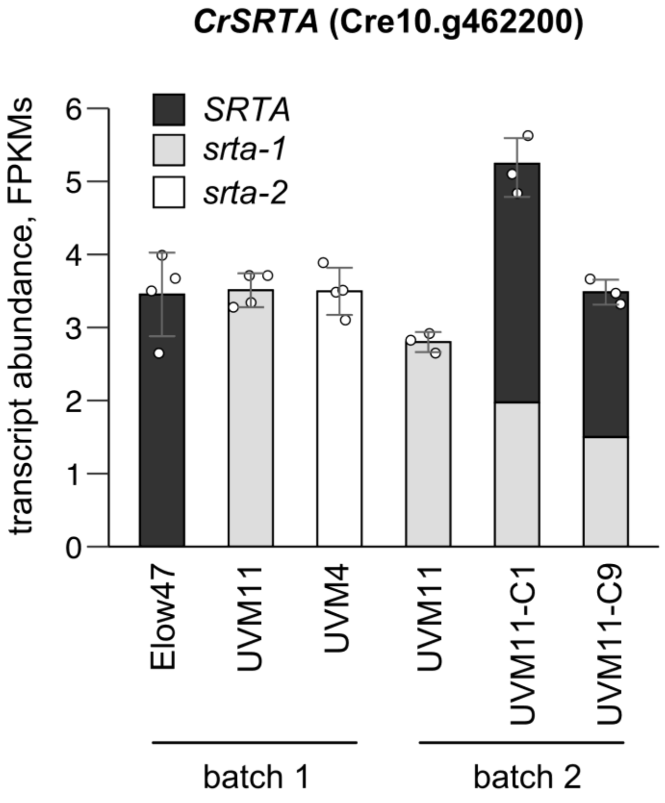

Supplementary Figure 13. Transcript abundance of *SRTA* as determined by RNAseq. The FPKMs for the gene are plotted for each of the three strains. Bars represent the mean, error bars indicate the standard deviation (n=4), and individual data points are shown as open circles. Source data are provided as a Source Data file.
