# Supplementary material for: An epigenetic gene silencing pathway selectively acting on transgenic DNA in the green alga Chlamydomonas
Source: Nat Commun. 2020 Dec 8;11:6269. doi: 10.1038/s41467-020-19983-4 (PMC7722844; doi:10.1038/s41467-020-19983-4)
Supplement: Supplementary file 3 — Description of Additional Supplementary Files [file 41467_2020_19983_MOESM3_ESM.pdf]

Description of Additional Supplementary Files

Supplementary Data 1:

Expression analysis of progeny (UVM11-CW x CC-2290). Analysis of YFP expression capacity of the progeny obtained from genetic crosses of UVM11-CW x CC-2290. Based on the strength of the YFP expression, the mapping population was selected (see Supplementary Data 2). Only offspring with at least 30% of all transformants analyzed showing strong YFP fluorescence were considered to have the mutation and were included in the mapping population.

Supplementary Data 2:

Segregants from genetic crosses of UVM11-CW x CC-2290 that showed strong YFP expression capacity (at least 30% of all transformants analyzed show YFP fluorescence) were considered to have the mutation and were included in the mapping population.

Supplementary Data 3:

Marker analysis of the mapping population (UVM11-CW x CC-2290). PCR markers on the 17 linkage groups were analyzed (http://www.chlamycollection.org/products/mapping-kits/), the allele identity was determined (by the size of the amplicon) and the recombination frequency was calculated for each marker.

Supplementary Data 4:

List of genes localized between markers CNA83 and PF25 on chromosome 10. Genes with putative chromatin-related functions according to the gene annotation of *Chlamydomonas reinhardtii* genome v5.5 (http://phytozome.jgi.doe.gov/) are highlighted in yellow. The four new markers (X1, X2, X3 and X4) that were created and are localized within the 3' UTRs of the genes Cre10.g461750, Cre10.g462200, Cre10.g464264 and Cre10.g464750, respectively, are also indicated.

Supplementary Data 5:

Variant loci identified by whole-genome sequence analysis. The list shows all variant loci identified in strains UVM4, UVM11, Elow47 and CC-4350. Variants with low-confidence base calls were excluded from the analysis.

Supplementary Data 6:

Differentially expressed genes that are up- or down-regulated in expression strains, complemented expression strains and the control strain identified by RNAseq. In the batch 1 analysis, the transcriptomes of UVM4 and UVM11 were compared to that of Elow47. In batch 2, the differentially expressed genes were identified from the comparison of the RNAseq datasets of the complemented strains UVM11-C1 and UVM11-C9 with the UVM11 mutant strain.

Supplementary Data 7:

Differentially expressed genes that are up-regulated in UVM4 and UVM11 and, additionally, are among the class of down-regulated genes in the complemented UVM11 strains compared to UVM11.

Supplementary Data 8:

Genome-wide survey of transposons in expression strains and control strains. The presence of nine different transposons in the genomes of UVM4, UVM11, the wild type-like strain Elow47 and the wild type cw15 arg- was determined by whole genome sequencing. Transposons that do not occur in all strains are indicated with an 'X'.
