# Supplementary material for: An epigenetic gene silencing pathway selectively acting on transgenic DNA in the green alga Chlamydomonas
Source: Nat Commun. 2020 Dec 8;11:6269. doi: 10.1038/s41467-020-19983-4 (PMC7722844; doi:10.1038/s41467-020-19983-4)
Supplement: Supplementary file 12 — Reporting Summary [file 41467_2020_19983_MOESM12_ESM.pdf]

nature research

Corresponding author(s): Ralph Bock

Last updated by author(s): Nov 1, 2020

Reporting Summary

Nature Research wishes to improve the reproducibility of the work that we publish. This form provides structure for consistency and transparency in reporting. For further information on Nature Research policies, see Authors & Referees and the Editorial Policy Checklist .

Statistics

For all statistical analyses, confirm that the following items are present in the figure legend, table legend, main text, or Methods section.

n/a	Confirmed	
☐	☒	The exact sample size (*n*) for each experimental group/condition, given as a discrete number and unit of measurement
☒	☐	A statement on whether measurements were taken from distinct samples or whether the same sample was measured repeatedly
☐	☒	The statistical test(s) used AND whether they are one- or two-sided<br>*Only common tests should be described solely by name; describe more complex techniques in the Methods section.*
☒	☐	A description of all covariates tested
☒	☐	A description of any assumptions or corrections, such as tests of normality and adjustment for multiple comparisons
☐	☒	A full description of the statistical parameters including central tendency (e.g. means) or other basic estimates (e.g. regression coefficient) AND variation (e.g. standard deviation) or associated estimates of uncertainty (e.g. confidence intervals)
☐	☒	For null hypothesis testing, the test statistic (e.g. *F*, *t*, *r*) with confidence intervals, effect sizes, degrees of freedom and *P* value noted<br>*Give P values as exact values whenever suitable.*
☒	☐	For Bayesian analysis, information on the choice of priors and Markov chain Monte Carlo settings
☒	☐	For hierarchical and complex designs, identification of the appropriate level for tests and full reporting of outcomes
☒	☐	Estimates of effect sizes (e.g. Cohen's *d*, Pearson's *r*), indicating how they were calculated

Our web collection on statistics for biologists contains articles on many of the points above.

Software and code

Policy information about availability of computer code

Data collection

NCBI (https://www.ncbi.nlm.nih.gov/)
Phytozome web page (Phytozome v10, v11 or v12.1; http://www.phytozome.net/)
Saccharomyces genome database (http://www.yeastgenome.org/)
HGNC (http://www.genenames.org/cgi-bin/genefamilies/set/937) database

Data analysis

Genome sequencing:
Phytozome v12.1 (http://phytozome.net)
BWA mem, version 0.7.5a-r405
Picard MarkDuplicates, version 1.85(1345) (http:// broadinstitute.github.io/picard)
Genome Analysis Toolkit (GATK), version 2.6-5-gba531bd
SnpEff version 4.3r
Transposon Analysis:
BWA mem, version 0.7.5a-r405
samtools (v 1.9-58-gbd1a409)
bedtools (v2.29.2) genomecov
IGV version 2.8.10. (http://software.broadinstitute.org/software/igv/UserGuide)
RNAseq:
Phytozome v12.1(http://phytozome.net)
RNA-STAR (v2.4.0j) with --alignIntronMax 3000
featureCounts program of Rsubread package (v1.12.6) within the R statistical computing platform (v4.0.1)
DESeq2 package (v1.18.1) in R with prcomp function
ggplot2 (v3.3.2)

plotMA function in DESeq2
apeglm method in R
VennDiagram package (v1.6.20) in R
cuffdiff (v2.2.1)
cummeRbund (v2.24.0) in R
general bioinformatics analyses:
(Phytozome v10, v11 or v12.1; http://www.phytozome.net/)
ModBase version r225 (https://modbase.compbio.ucsf.edu/modweb/)
Chimera version 1.12 (http://www.cgl.ucsf.edu/chimera).
ClustalW software version 2.0 (https://www.ebi.ac.uk/Tools/msa/clustalw2/)
MEGAX software version MEGA_X_10.0.5 (https://www.megasoftware.net/)

For manuscripts utilizing custom algorithms or software that are central to the research but not yet described in published literature, software must be made available to editors/reviewers. We strongly encourage code deposition in a community repository (e.g. GitHub). See the Nature Research guidelines for submitting code & software for further information.

Data

Policy information about availability of data

All manuscripts must include a data availability statement. This statement should provide the following information, where applicable:

- Accession codes, unique identifiers, or web links for publicly available datasets
- A list of figures that have associated raw data
- A description of any restrictions on data availability

Genome sequencing reads are available from the NCBI Sequence Read Archive under accession numbers SRR1797981 (cw15arg-), SRR6872092 (Elow47), SRR6872091 (UVM4) and SRR6872090 (UVM11). The RNAseq data, including raw reads and FPKM expression tables, were deposited in the NCBI Gene Expression Omnibus (GEO) database under accession GSE128981. Raw data of the ChIP experiment are available from the corresponding author upon request. The authors declare that all other data supporting the findings of this study are available within the paper and its suplementary information files.

Field-specific reporting

Please select the one below that is the best fit for your research. If you are not sure, read the appropriate sections before making your selection.

☒ Life sciences ☐ Behavioural & social sciences ☐ Ecological, evolutionary & environmental sciences

For a reference copy of the document with all sections, see nature.com/documents/nr-reporting-summary-flat.pdf

Life sciences study design

All studies must disclose on these points even when the disclosure is negative.

Sample size

Three independent srta mutant strains were analyzed (srta-1, srta-2, srta-3).

[Rationale:
The srta mutant strains srta-1 and srta-2 were isolated from the mutagenesis screen. The third mutant line, srta-3, was obtained from the Chlamydomonas insertion mutant library, and represents the only insertion line available for this locus.]

ChIP-qPCR analysis was performed using pools of at least 1200 independent YFP transgenic lines for each strain (i.e., antibiotic-resistant colonies obtained after transformation with the YFP-containing transformation vector were pooled).

[Rationale:
It was shown previously that, when more than 240 transformants harboring the ble gene were pooled, variation in ble expression between individual transformants was averaged out (PMID: 17993574).]

The mapping population was composed of 48 segregants, all of them showing strong transgene expression.

[Rationale: Size of mapping population was chosen according to the suggestions as given in the map based cloning strategy for Chlamydomonas published earlier (PMID: 15665247).]

To assess the transgene expression capacity of each individual strain, on average 20 colonies grown on selection medium (after transformation with a YFP cassette-containing transformation vector) were randomly picked and screened for YFP expression/ fluorescence intensity. From previous results, it has been known that approximately 50% of the randomly picked colonies contain the full-length transgene cassette integrated into the genome (PMID: 19036032, 26402748).
For the complementation analysis, 26 and 24 individual SRTA transformants carrying the full-length SRTA wild-type allele of UVM4 and UVM11, respectively, were analyzed. In addition, for each strain (UVM4 and UVM11), 18 transgenic clones that had been obtained after transformation with the SRTA-FLAG-containing transformation vector were analyzed for complementation.
For RNAseq analysis, two batches were analyzed. Batch 1 was composed of strains UVM4, UVM11 and Elow47 with 4 biological replicates each. Batch 2 was composed of strain UVM11 and two complemented lines of UVM11 expressing the SRTA wild type allele (UVM11-C2 and UVM11-C9) with 3 biological replicates for each strain.

Data exclusions	No data were excluded.
Replication	ChIP experiments were performed using a pool of at least 1200 independent transformant colonies for each strain followed by a qPCR analysis with three technical replicates per sample. Three experiments each were done on two independent pools of at least 1200 transformants. All attempts at replication were successful.<br>RNAseq analysis was performed with four biological replicates for each strain included in batch 1 (UVM4, UVM11, and Elow47) and with 3 biological replicates for each strains included in batch 2 (UVM11, UVM11-C2 and UVM11-C9). All attempts at replication were successful
Randomization	All transgenic algal clones analyzed were picked randomly.
Blinding	Blinding was not applicable because the study does not involve animals and/or human research participants.

Reporting for specific materials, systems and methods

We require information from authors about some types of materials, experimental systems and methods used in many studies. Here, indicate whether each material, system or method listed is relevant to your study. If you are not sure if a list item applies to your research, read the appropriate section before selecting a response.

Materials & experimental systems

n/a	Involved in the study
☐	☒ Antibodies
☐	☒ Eukaryotic cell lines
☒	☐ Palaeontology
☒	☐ Animals and other organisms
☒	☐ Human research participants
☒	☐ Clinical data

Methods

n/a	Involved in the study
☒	☐ ChIP-seq
☒	☐ Flow cytometry
☒	☐ MRI-based neuroimaging

Antibodies

Antibodies used	anti-H3 (ab1791; Abcam)<br>anti-H3ac (06-599; Merck)<br>anti-H3K4me1 (ab8895; Abcam)<br>anti-H3K4me2 (07-030; Merck)<br>anti-H4ac (K5, 8, 12, 16; AHP418; Biorad)<br>diacetyl H3K9 and H3K14 (Upstate 06-599)<br>tetra-acetyl H4K5, H4K8, H4K12, and H4K16 (Upstate 06-866)<br>monomethylated H3K9 (ab9045; Abcam).<br>anti-GFP (632381; Clontech)<br>goat anti-rabbit secondary antibody (170-6515; Biorad)<br>goat anti-mouse secondary antibody (AS111772; Agrisera)
Validation	anti-H3 (ab1791; Abcam) - PMID: 22050920; Abcam:"This product Rabbit Anti-Histone H3 antibody - Nuclear Loading Control and ChIP Grade. [..] Predicted to work with: a wide range of other species, Mammals" (https://www.abcam.com/histone-h3-antibody-nuclear-loading-control-and-chip-grade-ab1791.html)<br><br>anti-H3ac (06-599; Merck) – PMID: 23585280; Merck: "Detect acetyl-Histone H3 with Anti-acetyl-Histone H3 Antibody (Rabbit Polyclonal Antibody), that has been shown to work in WB, ICC, ChIP, ChIP-seq. [..] Demonstrated to react with human, mouse, and rat. Broad species cross-reactivity expected based on sequence similarity." (https://www.merckmillipore.com/DE/de/product/Anti-acetyl-Histone-H3-Antibody,MM_NF-06-599)<br><br>anti-H3K4me1 (ab8895; Abcam) - PMID: 23585280; Abcam: "This product Rabbit Anti-Histone H3 (mono methyl K4) antibody - ChIP Grade. [..] Predicted to work with: Cow, Indian muntjac, Plants, Mammals." (https://www.abcam.com/histone-h3-mono-methyl-k4-antibody-chip-grade-ab8895.html)<br><br>anti-H3K4me2 (07-030; Merck): PMID: 23585280; Merck: "Use Anti-dimethyl-Histone H3 (Lys4) Antibody (rabbit polyclonal antibody) is published and validated in ChIP, DB, ICC, IF, WB, PIA, ChIP-seq to detect dimethyl-Histone H3 (Lys4) also known as H3K4me2. [..] Human and tetrahymena. Broad species cross-reactivity is expected." (https://www.merckmillipore.com/DE/de/product/Anti-dimethyl-Histone-H3-Lys4-Antibody,MM_NF-07-030)<br><br>anti-H4ac (K5, 8, 12, 16; AHP418; Biorad) - PMID: 23585280; Biorad: "Use Anti-acetyl-Histone H4 Antibody (Rabbit Polyclonal Antibody) has been published and validated in ChIP, WB, ICC to detect acetyl-Histone H4 also known as H4 histone family member A, histone 1-H4a. [..]Human and Tetrahymena. Other species not tested, but expected to cross-react since Histone H4 is well conserved." (https://images.bio-rad-antibodies.com/datasheets/datasheet-AHP418.pdf)

monomethylated H3K9 (ab9045; Abcam) - PMID: 23585280; Abcam: “This product Rabbit Anti-Histone H3 (mono methyl K9) antibody - ChIP Grade. [...] Reacts with: Mouse, Rat, Cow, Human, Xenopus laevis, Arabidopsis thaliana, Indian muntjac, Schizosaccharomyces pombe.“ https://www.abcam.com/histone-h3-mono-methyl-k9-antibody-chip-grade-ab9045.html

anti-GFP (632381; Clontech) - PMID: 30150628; Clontech: "This antibody recognizes native and denatured forms of wild-type GFP, GFPuv, AcGFP, EGFP, destabilized EGFP variants, EBFP, EYFP, ECFP, AcGFP, and both N- and C-terminal fusion proteins containing these GFP variants in bacterial and mammalian cell lysates." (https://www.takarabio.com/assets/documents/Certificate%20of%20Analysis/632380-632381-070313.pdf)

goat anti-rabbit secondary antibody (170-6515; Biorad), - PMID: 17435233; Biorad: “Blotting Grade Goat Anti-Rabbit IgG (H + L) (Human IgG Adsorbed) Horseradish Peroxidase Conjugate.” http://www.bio-rad.com/webroot/web/pdf/lsr/literature/LIT418.pdf

goat anti-mouse secondary antibody (AS111772; Agrisera) - PMID: 30150628; Agrisera: "Goat anti-mouse IgG (H&L) is a secondary antibody conjugated to HRP, which binds to mouse IgG (H&L) in immunological assays." (https://www.agrisera.com/en/artiklar/goat-anti-mouse-igg-hl-hrp-conjugated.html)

diacetyl H3K9 and H3K14 (Upstate 06-599, now Merckmillipore) - PMID: 22050920; Merck: " This antibody has been shown to work for chIP using HeLa extracted chromatin [...]Detect acetyl-Histone H3 with Anti-acetyl-Histone H3 Antibody (Rabbit Polyclonal Antibody), that has been shown to work in WB, ICC, ChIP, ChIP-seq." (https://www.merckmillipore.com/DE/de/product/Anti-acetyl-Histone-H3-Antibody,MM_NF-06-599?ReferrerURL=https%3A%2F%2Fwww.bing.com%2F&bd=1#overview)

tetra-acetyl H4K5, H4K8, H4K12, and H4K16 (Upstate 06-866, now MerckMillipore) - PMID: 22050920; Millipore: "Use Anti-acetyl-Histone H4 Antibody (Rabbit Polyclonal Antibody) has been published and validated in ChIP, WB, ICC to detect acetyl-Histone H4 also known as H4 histone family member A, histone 1-H4a." (https://www.merckmillipore.com/DE/de/product/Anti-acetyl-Histone-H4-Antibody,MM_NF-06-866?ReferrerURL=https%3A%2F%2Fwww.bing.com%2F&bd=1)

Eukaryotic cell lines

Policy information about cell lines

Cell line source(s)	Cell lines were obtained from the Chlamydomonas Stock Center (http://chlamycollection.org/strains/). Chlamydomonas insertion mutants were obtained from the Chlamydomonas mutant library (CLiP, https://www.chlamylibrary.org/).The generation of the mutant strains UVM4 and UVM11 and the Elow47 control strain has been described previously (PMID: 19036032). Details about new strains that were generated by crossing are given within the paper.
Authentication	Authentication of the insertion mutants was performed according to the instructions given by the Chlamydomonas mutant library (https://www.chlamylibrary.org/files/Instructions%20on%20PCRs%20to%20check%20the%20insertion%20site.pdf) using Southern blot and PCR analysis.
Mycoplasma contamination	The cell lines used are algal strains and, therefore, were not tested for mycoplasma contamination.
Commonly misidentified lines (See ICLAC register)	No commonly misidentified lines were used.
