# Supplementary material for: An epigenetic gene silencing pathway selectively acting on transgenic DNA in the green alga Chlamydomonas
Source: Nat Commun. 2020 Dec 8;11:6269. doi: 10.1038/s41467-020-19983-4 (PMC7722844; doi:10.1038/s41467-020-19983-4)
Supplement: Supplementary file 2 — Peer Review File [file 41467_2020_19983_MOESM2_ESM.pdf]

Reviewers' comments:

Reviewer #1 (Remarks to the Author):

In this study, Neupert et al. identified the molecular basis of the strong transgene suppression in the reference alga Chlamydomonas reihardtii. Through a series of well-designed and executed experiments, the authors mapped a mutation, which resulted in transgene de-repression by transforming the individual segregants with a reporter gene. They identified a Sir2-type histone deacetylase (Cre10.g462200 encoding SRTA) as an underlying epigenetic factor of transgene suppression. This finding was verified by complementation experiments and characterization of an insertion mutant line. This work is of great interest to alga and plant biologists who suffer from gene silencing that is associated with transgenesis. However, the manuscript is weak in providing any mechanistic insight in the role of SRTA in genome integrity and protection.

Major issues:

Pull-down experiment with the FLAG-tagged complementing line should be performed to identify the molecular target(s) of SRTA. We do not know whether SRTA directly interacts with histones or with other proteins (non-histone effect as shown in the case of many histone deacetylases).

The effect of srta on gene expression should be investigated with clean mutant and complemented lines, not with UVM4 and UVM11, which derived from a UV mutagenesis screen and harbour hundreds of mutations.

Minor issues:

Page 5, ChIP experiments should be introduced/described more clearly. It is confusing to read the first paragraph without knowing the rationale behind the antibody selection.

Page 6, The authors claim that "epigenetic regulation of transposon activity is not affected by the mutation in the transgene suppression pathway". However, they investigated the activity of two mobile genetic elements (TOC1 and Gulliver) by genomic southern blotting. This is not enough to make such a claim. Instead, they should analyze the UVM4 and UVM11 whole genome sequencing data (page 8) for new integration sites.

Page 6, "sensitivity to DNA-damaging agents was unaffected". Since only Zeocin was tested, this sentence should be modified accordingly.

Figure 4b needs further clarification.

Page 15, " SRTA may act preferentially on DNA that is naked and not yet covered by chromatin proteins". Discussion is too speculative; no experiment was performed to suggest this.

Most viruses (RNA and DNA) do not integrate to the host genome. Thus the authors claim that SRTA might be involved in antiviral defense is implausible.

Reviewer #2 (Remarks to the Author):

Chlamydomonas is notoriously difficult to stably express transgenes and this has continuously plagued the field. UVM4 and UVM11 are UV mutants that overcome some of these difficulties but their molecular mechanism was unknown. Here Neupert et al. identify the causative mutations for UVM4

and UVM11 and reveal a mechanism for their ability to express transgenes. These mutants have alterations to a sirtuin-type histone deacetylase that results in altered histone acetylation status of transgenic DNA. The authors present data that suggests that incoming transgenic DNA is inactivated via assembly of repressive chromatin on that transgene, without major changes to the endogenous transcriptome. These results will be of much interest to the research community as these lines (UVM4 and UVM11) are widely used and the mechanisms of how Chlamydomonas silences transgenes is not completely understood. Additionally, this work provides a possible route to help increase transgene expression in recalcitrant algae or organisms through inactivation of histone deacetylase. This work will influence thinking in the field. The work presented is convincing, is presented in a way that could be reproduced by others in the field, and utilizes appropriate statistical methods.

Minor suggestions/comments
The data presented in Fig. 5 of silencing of already existing transgenes suggests that SRTA also may work outside of the proposed mechanism of newly inserted naked transgenic DNA being recognized and then inactivated by repressive chromatin. More discussion (pg 15) of how this could work (potential mechanism) and how this fits in with the other proposed mechanisms would help strengthen the discussion.

The authors suggest that SRTA inactivation could be used as a tool to enhance transgene expression. Several histone deacetylase inhibitor drugs exist. Were any of these evaluated to see if they can improve YFP expression in WT algae? If this approach is successful it could allow for more rapid expression of transgenes in WT backgrounds of Chlamydomonas but also potentially other organisms where mutants in this gene do not exist.

In the last two paragraphs of the discussion the sentence about use of UVM strains as a tool seems out of place within the discussion of environmental DNA. It may make a stronger message to rearrange these ideas.

Defining 'supertransformed' on pg 10 may help some readers who are not familiar with this term.

Reviewer #3 (Remarks to the Author):

In their manuscript, Neupert et al. pursue the genetic components that are responsible for the silencing of transgenes in the green alga Chlamydomonas reinhardtii. This species is known in molecular biology for its unwillingness to express transgenic constructs, and researchers commonly use mutated strains with reduced silencing capacity when analyzing transgenes in this species. However, the genetic basis underlying the properties of those mutants, and hence of the transgene silencing, have never been investigated.

Neupert and colleagues show that histone occupancy at transgenes is low in transgenic regions, and that several histone modifications show altered frequencies compared to endogenous genes, which are often reverted in the silencing-suppressed mutants. Most notably, histone H3 acetylation is increased in the mutants at the transgene TSS, while repressive H3 methylation is decreased. When mapping the causal mutation of two mutant strains, the authors in both cases identify a sirtuin2-type histone deacetylase (SRTA). Through additional insertion lines and complementation assays, the authors convincingly show that SRTA is the essential to the transgene silencing. Additional experiments indicate that SRTA might act solely on new incoming transgenes and might not affect already established transgene copies in the genome. Transcriptome analyses do not reveal a more general function of SRTA.

Overall, the study design is elegant. The reporter system and the crosses done in order to map the

causal gene are convincing. As explained below, the data on the chromatin accessibility needs better visualization and more detail in the representation. I was wondering why the authors did not include a chromatin accessibility profile via ATAC-seq in their study, which would have delivered more reliable data and would at the same time have allowed a genome-wide profiling of accessibility in the srta mutants.

The conclusion that "naked DNA is the preferred target of SRTA-mediated silencing", which is the main novelty claim of the paper, remains highly speculative at this point, as the experiments performed in this study do not allow to make this statement with certainty, and additional experiments are necessary to test this hypothesis. The speculation that the SRTA-based silencing of transgenes might be an evolutionary alternative to the CRISPR/Cas9 system – catchy as it may be – seems far-fetched and premature.

Minor comments:

- Page5: "…chromatin immunoprecipitation (ChIP) experiments were conducted with pools of independent YFP transformants…". Without specifying the target of the ChIP, this sentence does not provide the necessary information.

- Page 5: what are "ChIP values"? This is not a term commonly used in the field.

- Fig. 1a: How many cells were analyzed per condition (n=?)?

- Fig. 1b: The legend is missing all kinds of information: What are the error bars? How many replicates were done? How were the data normalized? Instead of error bars, these graphs would be much more informative with individual data points included, or as violin plots. As is, the differences could potentially result from few outlier replicates driving the mean.

- If the transcriptional effects observed in the transcriptome data of the two mutant strains might be – as the authors conclude – unspecific and related to additional mutations in these strains, why not perform a transcriptome analysis on the T-DNA insertion line, which should have a much lower mutation load?

We would like to thank the three reviewers for their thoughtful comments on our manuscript. As you will see from the point-by-point response below, we have addressed their criticisms in our revised manuscript. We provide a large amount of additional experimental data, including (i) RNAseq datasets demonstrating that SRTA is not involved in the expression of endogenous genes in the genome, (ii) a genome-wide analysis of transposable elements to exclude involvement of SRTA in the suppression of transposons, and (iii) tests with several chemical inhibitors of HDACs to determine their effects on transgene expression and chromatin modification.

Below please find a point-by-point response to the Reviewers' suggestions (with our response in red):

Reviewer #1:

In this study, Neupert et al. identified the molecular basis of the strong transgene suppression in the reference alga Chlamydomonas reihardtii. Through a series of well-designed and executed experiments, the authors mapped a mutation, which resulted in transgene de-repression by transforming the individual segregants with a reporter gene. They identified a Sir2-type histone deacetylase (Cre10.g462200 encoding SRTA) as an underlying epigenetic factor of transgene suppression. This finding was verified by complementation experiments and characterization of an insertion mutant line. This work is of great interest to alga and plant biologists who suffer from gene silencing that is associated with transgenesis. However, the manuscript is weak in providing any mechanistic insight in the role of SRTA in genome integrity and protection.

Major issues:

Pull-down experiment with the FLAG-tagged complementing line should be performed to identify the molecular target(s) of SRTA. We do not know whether SRTA directly interacts with histones or with other proteins (non-histone effect as shown in the case of many histone deacetylases).

We agree with the Reviewer that it would be desirable to identify proteins that SRTA interacts with. As suggested, we attempted to isolate putative interaction partners by co-immunoprecipitation experiments. Unfortunately, the anti-FLAG antibody showed strong cross-reactions with many endogenous proteins, thus precluding the specific pull-down of the tagged SRTA and its possible interaction partners. We are currently constructing new complementation constructs (with alternative epitope tags), but it will take a long time before even preliminary results will be available. Interactions with histones are likely to be transient and difficult to capture without crosslinking, and we therefore expect that considerable optimization efforts will be required to obtain reliable co-IP results.

The effect of srta on gene expression should be investigated with clean mutant and complemented lines, not with UVM4 and UVM11, which derived from a UV mutagenesis screen and harbour hundreds of mutations.

A clean insertion mutant is currently not available (see R#3, Minor point 5). Therefore, as suggested by the Reviewer, we have used two complemented strains of the UVM11 mutant and compared their transcriptomes with those of the UVM mutants and the control strains (Fig. 6). The data showed very clearly that there are no significant effects of SRTA on the expression of endogenous genes in the genome. The results of this large comparative transcriptomic analysis are described on p.14-17 of the revised manuscript and discussed in the Discussion section of the manuscript.

Minor issues:

Page 5, ChIP experiments should be introduced/described more clearly. It is confusing to read the first paragraph without knowing the rationale behind the antibody selection.

The antibody selection is explained on p.5 of the revised manuscript, and the ChIP experiments are now explained in much more detail (p.5-7). We also added more information to the legend to Fig.1 and the Methods section.

Page 6, The authors claim that “epigenetic regulation of transposon activity is not affected by the mutation in the transgene suppression pathway”. However, they investigated the activity of two mobile genetic elements (TOC1 and Gulliver) by genomic southern blotting. This is not enough to make such a claim. Instead, they should analyze the UVM4 and UVM11 whole genome sequencing data (page 8) for new integration sites.

As suggested by the Reviewer, we have analyzed the copy numbers and locations of transposons in the resequenced genomes. The data confirm that the suppression of transposon activity is not affected in the *srta* mutants. The data are presented in Table 1, described in the Results section on p.17-18 and discussed in the Discussion section in relation to genome stability and SRTA function.

Page 6, “sensitivity to DNA-damaging agents was unaffected”. Since only Zeocin was tested, this sentence should be modified accordingly.

Done.

Figure 4b needs further clarification.

We have added a sentence explaining the four complementation classes and their relationship to the total complementation efficiency.

Page 15, “ SRTA may act preferentially on DNA that is naked and not yet covered by chromatin proteins”. Discussion is too speculative; no experiment was performed to suggest this.

We have toned down the naked DNA hypothesis, but we feel that we should provide the reader with a reasonable hypothesis, how the SRTA-mediated transgene silencing might work and what could be its function in the wild type. Having excluded a role in expression of endogenous gene and in the control of transposons, the transgene-specific action of the silencing pathway is best explained by a protective function from potentially harmful environmental DNA. In the Discussion section of the revised manuscript, we make clear that this is currently only a hypothesis that is consistent with all our data, but that further experiments will be needed to ultimately confirm it.

Most viruses (RNA and DNA) do not integrate to the host genome. Thus the authors claim that SRTA might be involved in antiviral defense is implausible.

We believe that the fact that most viruses do not integrate into the genome is actually consistent with our speculation that SRTA could be involved in antiviral defense: Our data suggest that, once the foreign DNA is integrated into the genome, it becomes largely insensitive to the action of SRTA, and we, therefore, propose that transgene inactivation by establishment of a repressive chromatin structure occurs prior to integration into the genome. This is now better explained in the Discussion section of the revised manuscript. However, in the revised ms, we also clearly state that this is just a hypothesis that will need to be tested by further experiments.

Reviewer #2:

Chlamydomonas is notoriously difficult to stably express transgenes and this has continuously plagued the field. UVM4 and UVM11 are UV mutants that overcome some of these difficulties but their molecular mechanism was unknown. Here Neupert et al. identify the causative mutations for UVM4 and UVM11 and reveal a mechanism for their ability to express transgenes. These mutants have alterations to a sirtuin-type histone deacetylase that results in altered histone acetylation status of transgenic DNA. The authors present data that suggests that incoming transgenic DNA is inactivated via assembly of repressive chromatin on that transgene, without major changes to the endogenous transcriptome. These results will be of much interest to the research community as these lines (UVM4 and UVM11) are widely used and the mechanisms of how Chlamydomonas silences transgenes is not completely understood. Additionally, this work provides a possible route to help increase transgene expression in recalcitrant algae or organisms through inactivation of histone deacetylase. This work will influence thinking in the field. The work presented is convincing, is presented in a way that could be reproduced by others in the field, and utilizes appropriate statistical methods.

Minor suggestions/comments

The data presented in Fig. 5 of silencing of already existing transgenes suggests that SRTA also may work outside of the proposed mechanism of newly inserted naked transgenic DNA being recognized and then inactivated by repressive chromatin. More discussion (pg 15) of how this could work (potential mechanism) and how this fits in with the other proposed mechanisms would help strengthen the discussion.

We have expanded a bit on the discussion of potential mechanisms, but tried to refrain from too much speculation (and also point out that more work is needed to clarify the mechanism and test the idea that incoming foreign DNA is the preferred target of SRTA action).

The authors suggest that SRTA inactivation could be used as a tool to enhance transgene expression. Several histone deacetylase inhibitor drugs exist. Were any of these evaluated to see if they can improve YFP expression in WT algae? If this approach is successful it could allow for more rapid expression of transgenes in WT backgrounds of Chlamydomonas but also potentially other organisms where mutants in this gene do not exist.

We have tested three different HDAC inhibitors that are commercially available: trichostatin, sirtinol and OSS_128167. None of them improved transgene expression in the wild type, nor did any of these inhibitors further boost transgene expression in the mutant strains. The data (along with analyses of histone acetylation levels) are presented in Supplementary Figures 10-12 and described in the text (p.13-14 of the revised ms). Possible explanations for the insensitivity of SRTA (and transgene expression efficiency) to these HDAC inhibitors are discussed.

In the last two paragraphs of the discussion the sentence about use of UVM strains as a tool seems out of place within the discussion of environmental DNA. It may make a stronger message to rearrange these ideas.

Done.

Defining ‘supertransformed’ on pg 10 may help some readers who are not familiar with this term.

Done.

Reviewer #3:

In their manuscript, Neupert et al. pursue the genetic components that are responsible for the silencing of transgenes in the green alga Chlamydomonas reinhardtii. This species is known in molecular biology

for its unwillingness to express transgenic constructs, and researchers commonly use mutated strains with reduced silencing capacity when analyzing transgenes in this species. However, the genetic basis underlying the properties of those mutants, and hence of the transgene silencing, have never been investigated.

Neupert and colleagues show that histone occupancy at transgenes is low in transgenic regions, and that several histone modifications show altered frequencies compared to endogenous genes, which are often reverted in the silencing-suppressed mutants. Most notably, histone H3 acetylation is increased in the mutants at the transgene TSS, while repressive H3 methylation is decreased. When mapping the causal mutation of two mutant strains, the authors in both cases identify a sirtuin2-type histone deacetylase (SRTA). Through additional insertion lines and complementation assays, the authors convincingly show that SRTA is the essential to the transgene silencing. Additional experiments indicate that SRTA might act solely on new incoming transgenes and might not affect already established transgene copies in the genome. Transcriptome analyses do not reveal a more general function of SRTA.

Overall, the study design is elegant. The reporter system and the crosses done in order to map the causal gene are convincing. As explained below, the data on the chromatin accessibility needs better visualization and more detail in the representation. I was wondering why the authors did not include a chromatin accessibility profile via ATAC-seq in their study, which would have delivered more reliable data and would at the same time have allowed a genome-wide profiling of accessibility in the srta mutants.

We agree with the Reviewer that ATACseq would be the method of choice to determine chromatin accessibility at the genome-wide level. Unfortunately, despite significant efforts in several labs, so far, the method has not been successfully adapted for *Chlamydomonas*. We, therefore, hope that our ChIP data, together with our new set of RNAseq analyses that convincingly show that nuclear gene expression is not altered in the mutants (Fig. 6 in the revised ms), are considered strong enough evidence to conclude that global chromatin structure and accessibility is unlikely to be disturbed in the mutants.

The conclusion that “naked DNA is the preferred target of SRTA-mediated silencing”, which is the main novelty claim of the paper, remains highly speculative at this point, as the experiments performed in this study do not allow to make this statement with certainty, and additional experiments are necessary to test this hypothesis. The speculation that the SRTA-based silencing of transgenes might be an evolutionary alternative to the CRISPR/Cas9 system – catchy as it may be – seems far-fetched and premature.

We have toned down the naked DNA hypothesis, but we feel that we should provide the reader with a reasonable hypothesis, how the SRTA-mediated silencing might work and what could be its function in the wild type. Having excluded a role in expression of endogenous gene and in the control of transposons, the transgene-specific action of the silencing pathway is best explained by a protective function from potentially harmful environmental DNA. In the Discussion section of the revised manuscript, we make clear that this is currently only a hypothesis that is consistent with all our data, but that further experiments will be needed to ultimately confirm it. We agree with the Reviewer that the analogy to CRISPR/Cas systems is currently entirely speculative, and have removed these statements from the revised ms.

Minor comments:

- Page5: “…chromatin immunoprecipitation (ChIP) experiments were conducted with pools of independent YFP transformants…”. Without specifying the target of the ChIP, this sentence does not provide the necessary information.

The ChIP experiments, the pooling strategy, the choice of the antibodies, and the genomic regions investigated are now explained in more detail (p.5-7). We also added more information to the legend to Fig.1 and the Methods section.

- Page 5: what are "ChIP values"? This is not a term commonly used in the field.

We agree and have replaced the term with a more precise description of what is meant.

- Fig. 1a: How many cells were analyzed per condition (n=?)?

This information has been added to the legend (≥20 colonies with ≥200 cells each were analyzed).

- Fig. 1b: The legend is missing all kinds of information: What are the error bars? How many replicates were done? How were the data normalized? Instead of error bars, these graphs would be much more informative with individual data points included, or as violin plots. As is, the differences could potentially result from few outlier replicates driving the mean.

Data normalization is now explained in the legend (and in the text), the error bars are explained, and, in addition, we show the individual data points, as requested by the Reviewer.

- If the transcriptional effects observed in the transcriptome data of the two mutant strains might be – as the authors conclude – unspecific and related to additional mutations in these strains, why not perform a transcriptome analysis on the T-DNA insertion line, which should have a much lower mutation load?

Although improved libraries of insertion mutants have recently become available for *Chlamydomonas*, the use of insertion mutants, unfortunately, is still much less straightforward than in *Arabidopsis*. The available insertion mutant for *SRTA* is not a clean mutant in that it suffers from a large genomic rearrangement related to the insertion, which is a common phenomenon in *Chlamydomonas*. Unfortunately, the rearrangement at the *SRTA* locus also involves a translocation with another chromosome (see description on p.11 and in Supplementary Figures 6-8), making it likely that the insertion mutant suffers from numerous secondary effects caused by other genes involved in the rearrangement. To address the point of the Reviewer, we therefore designed an alternative experiment that we believe is more rigorous and informative than a transcriptome analysis of the insertion mutant. We used two complemented strains of the UVM11 mutant and compared their transcriptomes with those of the UVM mutants and the control strains (Fig. 6). The data showed very clearly that there are no significant effects of SRTA on the expression of endogenous genes in the genome. The results of this large comparative transcriptomic analysis are described on p.14-17 of the revised manuscript, represented in Fig. 6, and discussed in the Discussion section of the manuscript.

We hope that our revised manuscript is now suitable for publication in *Nature Communications*.

We apologize for the long time it took to submit this revision. In addition to the extensive experimental work that needed to be carried out, the Covid-19 pandemic, unfortunately, has caused substantial additional delays.

Reviewer #1 (Remarks to the Author):

The revised manuscript addressed most of my concerns. I am happy with the changes made to the manuscript.

Attila Molnar

Reviewer #2 (Remarks to the Author):

The additional experiments and updates to the text have helped to clarify and strengthen this version of the manuscript. These results will be of much interest to the research community, even if the exact molecular mechanism of SRTA is not completely determined at this time. There is a significant amount of data in this paper which will be extremely informative to the community and will help catalyze future research on transgene silencing in this and other organisms.

Minor:
It is somewhat disappointing that the HDAC inhibitors were not effective at improving transgene expression as this could have been a major applied finding of this work. Would adding the inhibitors to cells before, during, and after transformation potentially result in an effect (if this is what experiment was conducted it should be clarified as this wasn't clear to me).

Table 1 – The data contained within is not properly described. What is the difference between 'Total' and "All strains"? Also, for the individual strains what do these small numbers represent? Is the new or moved transposons?

line 460 (euchromatic?)

Reviewer #3 (Remarks to the Author):

In the revised version of their manuscript, the authors have addressed the criticisms and questions that I raised in the initial round of reviews. Most importantly, they included extensive RNA-seq analysis of the mutants and complemented lines, which provides strong support for their findings and interpretations. The authors also toned down some of the more speculative claims. Overall, I have no further comments and recommend the manuscript for publication.

Below please find a point-by-point response to the Reviewers' comments (with our response in red):

Reviewer #1:

No criticisms.

Reviewer #2:

Minor:
It is somewhat disappointing that the HDAC inhibitors were not effective at improving transgene expression as this could have been a major applied finding of this work. Would adding the inhibitors to cells before, during, and after transformation potentially result in an effect (if this is what experiment was conducted it should be clarified as this wasn't clear to me).

This has been clarified in the revised manuscript: The inhibitors were tested both before and after transformation, and in both cases, had no effect.

Table 1 – The data contained within is not properly described. What is the difference between 'Total' and "All strains"? Also, for the individual strains what do these small numbers represent? Is the new or moved transposons?

We have revised the labeling of the columns in this table to make those differences clearer.

line 460 (euchromatic?)

The question mark was removed.

Reviewer #3:

No criticisms.
